# Primary Cilia Mediate Diverse Kinase Inhibitor Resistance Mechanisms in Cancer

**DOI:** 10.1016/j.celrep.2018.05.016

**Published:** 2018-06-08

**Authors:** Andrew D. Jenks, Simon Vyse, Jocelyn P. Wong, Eleftherios Kostaras, Deborah Keller, Thomas Burgoyne, Amelia Shoemark, Athanasios Tsalikis, Maike de la Roche, Martin Michaelis, Jindrich Cinatl, Paul H. Huang, Barbara E. Tanos

**Affiliations:** 1 Division of Cancer Therapeutics, The Institute of Cancer Research, 237 Fulham Road, London SW3 6JB, UK; 2 Division of Molecular Pathology, The Institute of Cancer Research, 237 Fulham Road, London SW3 6JB, UK; 3 FILM, Sir Alexander Fleming Building, South Kensington Campus, Imperial College London, Exhibition Road, London SW7 2AZ, UK; 4 UCL Institute of Ophthalmology, London, UK; 5 Imperial College London, London, UK Electron Microscopy Department, Royal Brompton and Harefield NHS Foundation Trust, London, UK; 6 CRUK-Cambridge Research Institute, Cambridge CB2 0RE, UK; 7 Industrial Biotechnology Centre and School of Biosciences, University of Kent, Canterbury, UK; 8 Institute of Medical Virology, Goethe University Frankfurt, Paul-Ehrlich-Strasse 40, 60596 Frankfurt am Main, Germany

**Keywords:** cilia, kinase inhibitor, resistance, Hedgehog pathway, FGFR

## Abstract

Primary cilia are microtubule-based organelles that detect mechanical and chemical stimuli. Although cilia house a number of oncogenic molecules (including Smoothened, KRAS, EGFR, and PDGFR), their precise role in cancer remains unclear. We have interrogated the role of cilia in acquired and *de novo* resistance to a variety of kinase inhibitors, and found that, in several examples, resistant cells are distinctly characterized by an increase in the number and/or length of cilia with altered structural features. Changes in ciliation seem to be linked to differences in the molecular composition of cilia and result in enhanced Hedgehog pathway activation. Notably, manipulating cilia length via Kif7 knockdown is sufficient to confer drug resistance in drug-sensitive cells. Conversely, targeting of cilia length or integrity through genetic and pharmacological approaches overcomes kinase inhibitor resistance. Our work establishes a role for ciliogenesis and cilia length in promoting cancer drug resistance and has significant translational implications.

## Highlights

• Cancer cells show increased ciliogenesis and cilia signaling upon becoming drug resistant
• Cilia length control is misregulated in drug-resistant cells
• Increasing cilia length is sufficient to confer drug resistance
• Targeting cilia or cilia signaling can overcome kinase inhibitor resistance

## Graphical Abstract

**Figure:**
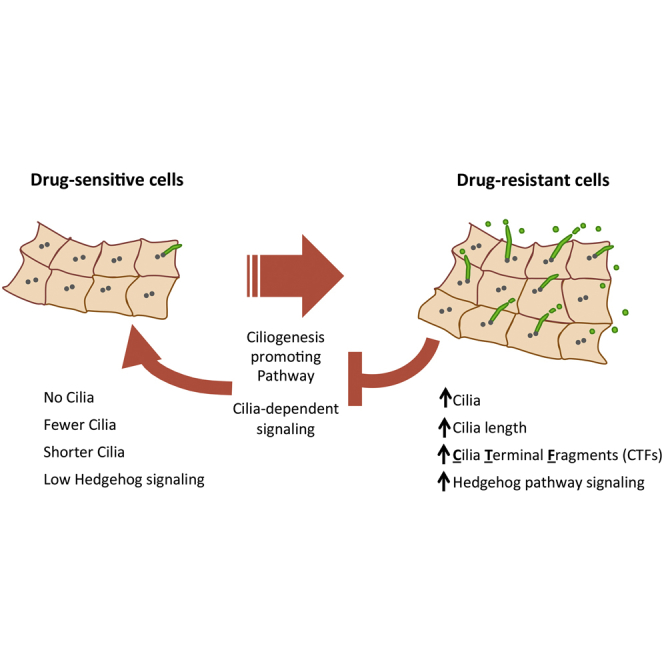

## Teaser

Jenks et al. demonstrate that enhanced ciliogenesis can facilitate resistance to a number of kinase inhibitors. Both acquired and *de novo* resistant cells show increases in cilia numbers and length and increased Hedgehog signaling. Targeting ciliogenesis or ciliary signaling overcomes kinase inhibitor resistance.

## Introduction

Primary cilia are microtubule-based sensory organelles that detect mechanical and chemical stimuli, and are formed by nearly all vertebrate cells (Garcia-Gonzalo and Reiter, 2012). These antenna-like organelles house a number of oncogenic molecules including Smoothened, KRAS (Lauth et al., 2010), epidermal growth factor receptor (EGFR), and platelet-derived growth factor receptor (PDGFR) (reviewed in Christensen et al., 2012). Although loss of cilia has been associated with the onset of malignancy in some human tumors (reviewed in Basten and Giles, 2013), in others, cilia appear to be necessary for cancer cell survival (Han et al., 2009, Wong et al., 2009, Li et al., 2016). In fact, depending on the nature of the driver oncogenic lesion, cilia can have opposing roles in tumorigenesis even in the same tumor type. For example, removal of cilia inhibited tumor growth in a mouse model of medulloblastoma (MB) driven by constitutively active Smoothened (SMO). However, cilia depletion in a GLI2-driven model of MB accelerated tumor growth (Han et al., 2009). Therefore, the role of cilia in cancer remains unclear and is likely to be context dependent. Furthermore, characterization of primary cilia in glioblastoma cells suggests that cancer-associated cilia may be structurally distinct (Moser et al., 2009, Sarkisian et al., 2014, Moser et al., 2014).

A number of cancer drugs inhibit proteins that have been shown to localize to cilia, such as EGFR and PDGFR (Christensen et al., 2012). These drugs (e.g., the EGFR inhibitor erlotinib) promote significant tumor regressions in appropriate patient populations (e.g., EGFR mutant non-small cell lung carcinoma patients). However, these responses are invariably followed by the emergence of lethal drug-resistant disease. Our understanding of the molecular mechanisms of drug resistance has facilitated the design and deployment of second-line therapies that can target drug-resistant tumors (Awada et al., 2015). However, the characterization of these mechanisms (particularly those that do not involve mutation of the drug target itself) has been limited, and specific to the individual target or drug. Thus, drug resistance remains the main obstacle in delivering long-lasting therapeutic benefit. The identification of cell biological processes that facilitate and support the emergence of drug resistance may provide new therapeutic opportunities with broad applicability.

In this study, we report that the number and length of primary cilia are upregulated both in *de novo* and acquired kinase inhibitor resistance (KIR). These changes are associated with distinct molecular and structural features at the cilium, including (1) failure to control cilia length, (2) increased Hedgehog pathway activation, and (3) cilia fragmentation. Cilia elongation via Kif7 knockdown is sufficient to increase survival in the presence of kinase inhibitors, thus suggesting that cilia elongation has a critical role in promoting drug resistance. Conversely, pharmacological targeting of ciliary pathways including fibroblast growth factor receptor (FGFR) and Hedgehog, or impairing ciliogenesis through downregulation of ciliary proteins can overcome resistance in all cell lines studied. Thus, we have uncovered a role for cilia in cancer that provides a rationale for targeting ciliogenesis as a broadly applicable strategy to overcome drug resistance.

## Results

### Ciliogenesis Is Upregulated in Isogenic Models of Acquired Drug Resistance

**Figure 1:**
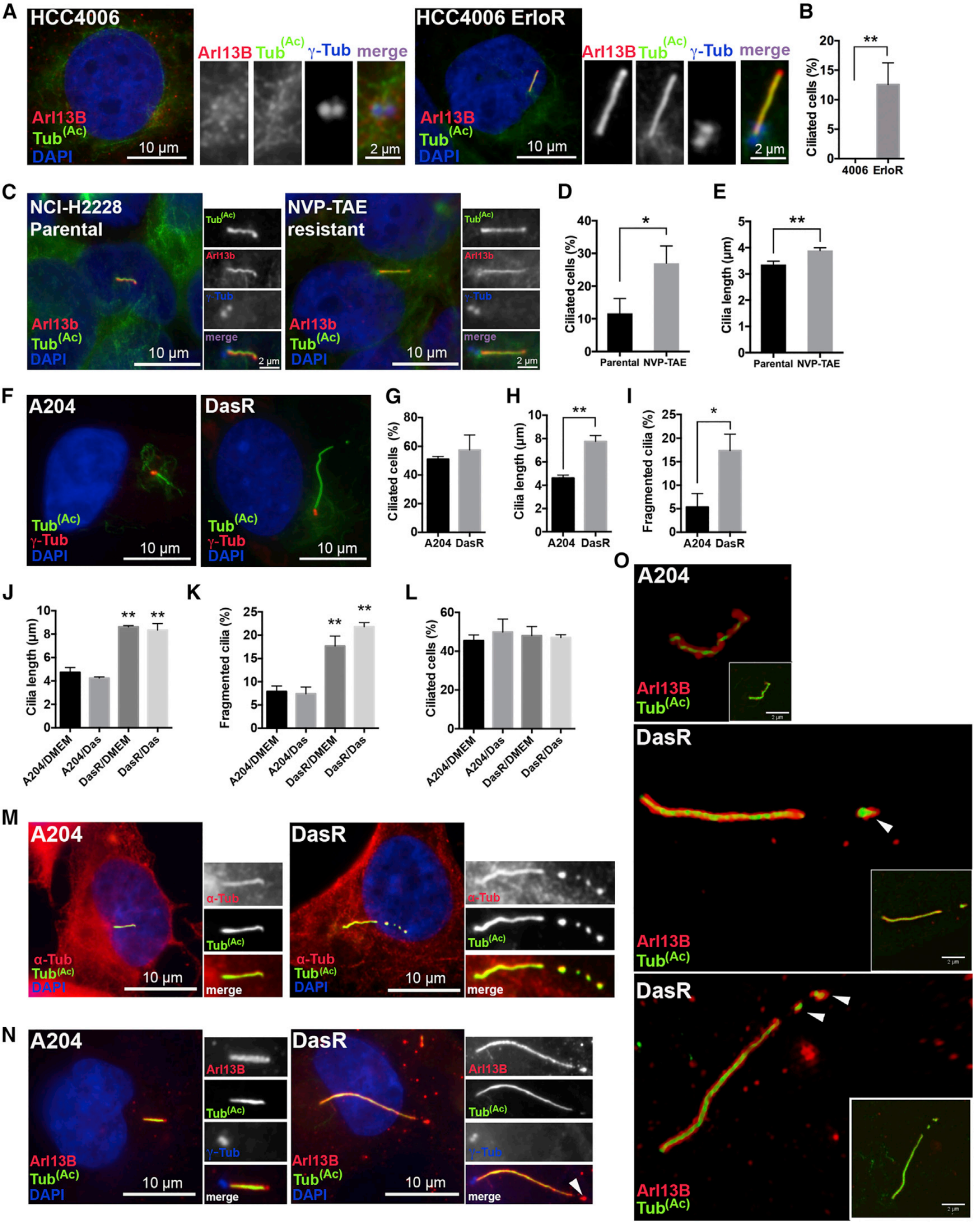
Acquired Resistance to Kinase Inhibitors in Human Cancer Cell Lines Is Associated with Increased Cilia Frequency, Cilia Length, and Cilia Tip Fragmentation (A) Control (left panels) or erlotinib-resistant (ErloR) (right panels) HCC4006 lung adenocarcinoma cells were serum starved for 48 hr to induce ciliogenesis, then fixed and stained with antibodies for acetylated tubulin (green) and Arl13B (red) to mark cilia, γ-tubulin (blue/inset) for centrioles, and DAPI (blue) to mark DNA. Note that primary cilia are absent in parental HCC4006 cells but are present in the erlotinib-resistant subline. (B) Quantification of experiment shown in (A). n = 300. Error bars represent SD. p < 0.005, unpaired t test. (C) Parental (left panels) or NVP-TAE684 (NVP-TAE)-resistant (right panels) NCI-H2228 lung adenocarcinoma cells were serum starved for 48 hr to induce ciliogenesis, and then fixed and stained with antibodies for acetylated tubulin (green) and Arl13B (red) to mark cilia, γ-tubulin (blue/inset) for centrioles, and DAPI (blue) to mark DNA. (D and E) Quantification of ciliated cells (D) and cilia length (E) shown in (C). n = 300 for (D) and n = 150 for (E). Error bars represent SD. p < 0.02 (D) and p < 0.005 (E), for an unpaired t test. Note that primary cilia were shorter in parental cells compared to the NVP-TAE684-resistant subline. (F) Rhabdoid tumor A204 cells (left panel) or a dasatinib-resistant (DasR) subline (right panel) were stained with acetylated tubulin to mark cilia (green), γ-tubulin (red), and with DAPI (blue). (G) Quantification of fraction of ciliated cells for the experiment shown in (F) (n = 300). (H and I) Quantification of cilia length (H) (n = 150) and cilia fragmentation (I) (n = 150) for the experiment shown in (F). Error bars represent the SD. p < 0.0007 for (H) and p < 0.011 for (I), unpaired t test. Note that DasR cells show increased cilia length and cilia fragmentation. (J–L) Quantification of primary cilia length (J), cilia fragmentation (K), and percentage of ciliated cells (L) for A204 or DasR cells grown with (Das) or without (DMEM) dasatinib for 48 hr, and then serum starved in the presence (Das) or absence (DMEM) of dasatinib for 48 hr. n = 150 cilia. The error bars represent the SD. p < 0.0001 for (J) and (K), Tukey’s multiple-comparison test, statistical significance calculated by comparing DasR/DMEM and DasR/Das to A204/DMEM and A204/Das. (M) A204 (left) or DasR (right) cells were serum starved to induce ciliogenesis, and then fixed and stained for α-tubulin (red) to mark all microtubules, acetylated tubulin (green) for cilia, and DAPI for DNA (blue). Note that α-tubulin is present along the entire cilium axoneme in both A204 and DasR cells and it follows cilia fragmentation in DasR cells (right). (N) A204 (left) or DasR cells (right) were stained for Arl13B to mark ciliary membranes (red), acetylated tubulin (green), γ-tubulin (blue/inset), and DAPI (blue). Arrow indicates cilia fragments marked by both acetylated tubulin and Arl13B in DasR cells. (O) 3D structured illumination images of A204 and DasR cilia; Arl13B is shown in red and acetylated tubulin in green. Note that, at this resolution, Arl13B signal surrounds acetylated tubulin. Arrows indicate budding fragments in DasR cells that contain membrane around them, suggesting an active budding event.

The role of primary cilia in human cancer is ill defined. Given the wide range of oncogenic proteins that are regulated by or localized to cilia (Christensen et al., 2012, Lauth et al., 2010), we hypothesized that changes in ciliogenesis could play a permissive role in the emergence of drug resistance. First, we examined EGFR-inhibitor resistance in the EGFR mutant non-small cell lung carcinoma (NSCLC) cell line HCC4006. We chose this model system because EGFR inhibitors are effective in the treatment of EGFR mutant lung cancer patients, but resistance to these drugs is inevitable (Tan et al., 2016). Furthermore, the mechanisms of drug resistance are still unknown for a large number of these patients. We examined ciliogenesis in these cells by staining for acetylated tubulin, a marker for cilia, or Arl13B, a marker specific for ciliary membranes (Caspary et al., 2007, Cevik et al., 2010). Interestingly, whereas control HCC4006 cells completely lacked primary cilia, erlotinib-resistant HCC4006 cells generated by chronic exposure to erlotinib (Saafan et al., 2016) (Figures S1A–S1D) showed robust staining for ciliary markers (Figure 1A).

We then asked whether changes in ciliogenesis could be seen in additional models of drug resistance, where the primary target was not EGFR. We examined ciliation in the EML4-ALK-fusion-positive lung cancer cell line NCI-H2228 (which is highly sensitive to the ALK inhibitor NVP-TAE684) and a drug-resistant derivative generated through chronic NVP-TAE684 exposure. Remarkably, NVP-TAE684 resistant NCI-H2228 cells showed increased cilia frequency and cilia length (Figures 1C–1E).

Next, we assessed ciliation in the rhabdoid tumor cell line A204 (which is exquisitely sensitive to the tyrosine kinase inhibitor dasatinib) and a dasatinib-resistant (DasR) subline generated through chronic drug exposure, which we recently characterized (Wong et al., 2016, Vyse et al., 2018) (Figure S1E). Notably, we found that compared to parental cells, DasR cells showed increased cilia length and tip fragmentation (Figures 1F–1I). These effects were neither acute nor transient, as short-term treatment with dasatinib did not promote these changes, and withdrawing dasatinib from DasR cells for several days did not revert the effect (Figures 1J–1L).

The changes in ciliogenesis observed in drug-resistant cells were seen in the absence (Figure 1) or presence of serum (Figure S2), suggesting they are independent of growth rates.

In total, we interrogated five isogenic models of acquired drug resistance (Table 1), of which only one (PC-9 lung adenocarcinoma cells) did not show alterations in cilia (Figure S2). In this model, however, cells with acquired resistance to the irreversible EGFR inhibitor afatinib showed a nearly complete biochemical insensitivity to the drug, consistent with the presence of a drug-binding-interfering mutation, a known and common mechanism of drug resistance (Wu et al., 2016). PC9 cells that were made resistant to erlotinib exhibited drug-resistant mitogen-activated protein kinase (MAPK) activity, which has also been described as a mechanism of acquired EGFR inhibitor resistance in both PC9 cells and in lung cancer patients (de Bruin et al., 2014).

**Table 1:** Cilia and Drug Resistance

Cell Lines	Drug Resistance	> Cilia Length	> Ciliated Cells	Figure
**Acquired Resistance**				

A204 DasR 1	Dasatinib	✔	–	Figures 1F–1I and S2A
A204 DasR 2	Dasatinib	✔	–	Figure S2A
H2228	NVP-TAE	✔	✔	Figures 1C–1E
HCC4006	Erlotinib	✔	✔	Figures 1A and 1B
PC9 Afatinib	Afatinib	–	–	Figures S2K and S2L
PC9 Erlotinib	Erlotinib	–	–	Figures S2K and S2L

***De Novo* Resistance**				

A549	Trametinib	✔	✔	Figures 5A, 5C, and 5D
H23	Trametinib	✔	✔	Figures S5B–S5D
H1792	Trametinib	–	✔	Figures S5E–S5G

**Acquired Chemoresistance**				

A549	Cisplatin	✔	–	Figures S2M–S2O
A549	Carboplatin	–	–	Figures S2M–S2O
A549	Vinflunine	✔	✔	Figures S2M–S2O

Summary of cilia changes observed in resistant cell lines.

A549 cells resistant to chemotherapeutic agents including cisplatin and vinflunine also showed increased ciliogenesis (Figures S2M–S2O). Thus, our models cover resistance to a range of clinically relevant therapeutic agents in several isogenic models. Table 1 summarizes the types of cilia changes identified in all models examined.

Cilia-derived vesicles have been shown to have important intercellular functions in tetrahymena and Chlamydomonas (Wang and Barr, 2016, Wood et al., 2013). However, cilia-derived fragments have not been described in cancer cells. We therefore set out to characterize the nature of the observed cilia fragmentation in drug-resistant cells by examining tubulin post-translational modifications. No major difference was observed in total tubulin acetylation (Figure S3A) or detyrosination (Figure S3B). Interestingly, we found that the extent of polyglutamylated tubulin, a modification shown to regulate microtubule stability (O’Hagan et al., 2011), showed slightly reduced intensity per unit length in DasR cells (Figure S3C).

Furthermore, staining with total α-tubulin together with acetylated tubulin clearly showed a discontinuous axonemal pattern in DasR cells compared to control cells (Figure 1M). These fragments were positive for the cilia marker Arl13B (Figure 1N). In fact, through super-resolution microscopy, we found that these fragments were completely surrounded by Arl13B-containing membrane (Figure 1O), suggesting that these are likely membrane-bound fragmented pieces of cilia.

### Cilia Length Control Is Involved in the Acquisition of Drug Resistance

**Figure 2:**
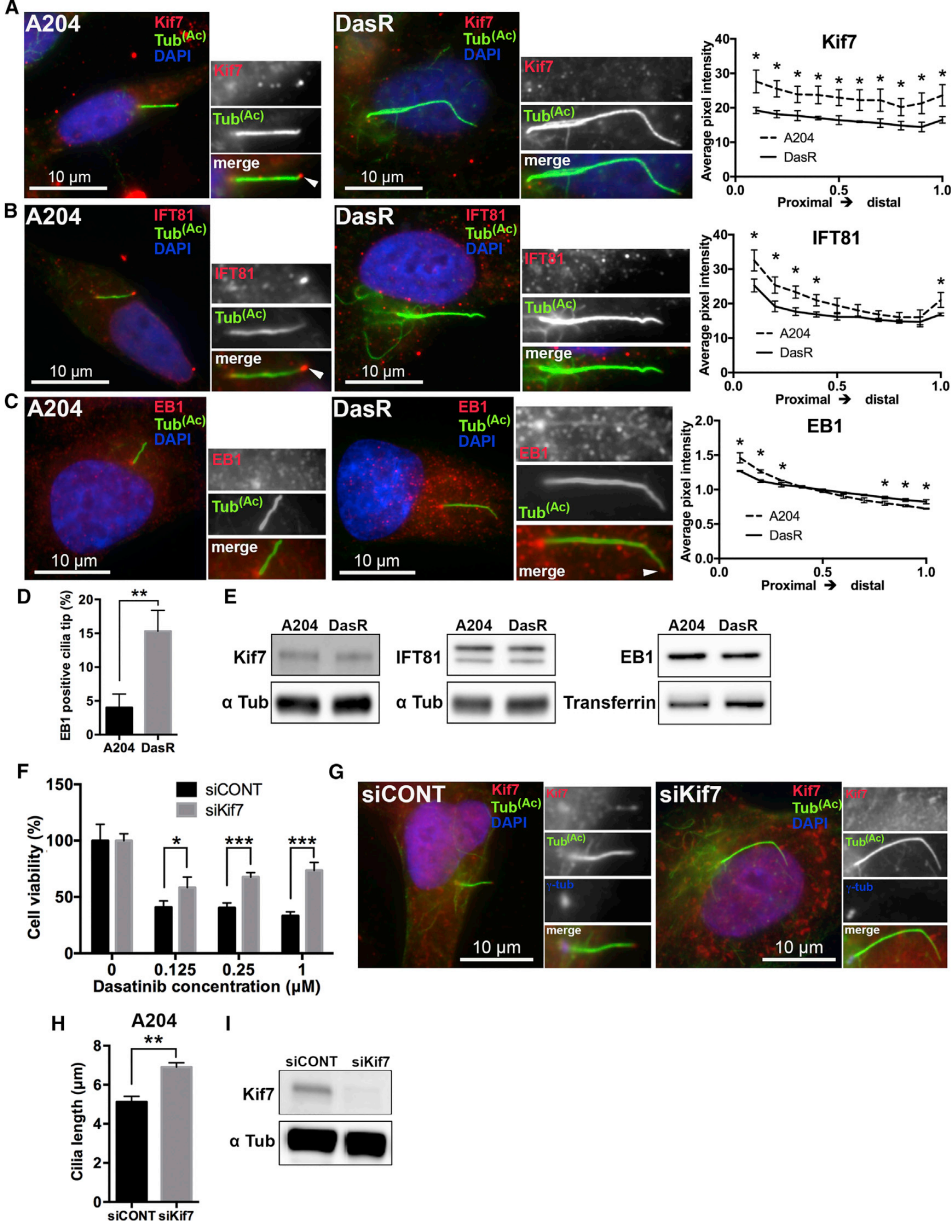
Cilia Length Control Is Critical for the Acquisition of Resistance (A) A204 (left panels) or DasR cells (right panels) were serum starved for 48 hr to induce ciliogenesis. After fixation, cells were stained with antibodies against acetylated tubulin (green), Kif7 (red), and with DAPI (DNA, blue). Kif7 staining along the length of the cilia was quantified as shown. Error bars (n = 150) represent SD. Kif7 p values (unpaired t test), proximal to distal: < 0.02 < 0.01, < 0.02, < 0.03, < 0.03, < 0.03, < 0.03, < 0.03, < 0.03, and < 0.02. (B) A204 (left panels) and DasR cells (right panels) treated as in (A), were stained with antibodies for acetylated tubulin (green), IFT81 (red), and with DAPI (blue). IFT81 staining along the length of the cilia was quantified as shown. Error bars (n = 150) represent SD. IFT81 p values (unpaired t test), proximal to distal: < 0.03, < 0.02, < 0.001, < 0.02, and < 0.04. Note that both Kif7 and IFT81 are present along the cilia and at the cilia tip in control A204 cells (arrows) but are absent in DasR cells. (C) A204 cells (left panels) or DasR (right panels) were stained with acetylated tubulin to mark cilia (green), EB1 (red), and with DAPI (blue). Note that EB1 is present at the cilia tip of DasR cells (arrow/inset) but not in parental A204 cells. Quantification of EB1 is shown. n = 150. Error bars represent SD. EB1 p values, proximal to distal: < 0.02, < 0.002, < 0.03, < 0.02, < 0.005, and < 0.006, for an unpaired t test. (D) EB1 was visually confirmed at the cilia tip of A204 and DasR cells. Chart shows quantification. n = 150. Error bars represent SD. p < 0.006, for an unpaired t test. (E) Western blots showing total protein levels of Kif7, IFT81, and EB1 (upper panels, indicated) and loading controls (lower panels) in A204 and DasR cells. (F) Cell viability (CellTiter-Glo) of A204 cells grown in the presence of vehicle (DMSO) or dasatinib (doses are indicated), transfected with either control siRNA or Kif7 siRNA (as indicated). Cell viability was normalized to DMSO-treated cells (n = 4). Error bars represent SD. p < 0.02 (0.125 μM), p < 0.0001 (0.25 μM), and p < 0.0001 (1 μM), unpaired t test. (G) A204 cells were serum starved for 48 hr to induce ciliogenesis, and then fixed and stained with antibodies for acetylated tubulin (green) and Kif7 (red), and with DAPI (blue). Note that A204 cells transfected with a Kif7 siRNA (siKif7) had increased cilia length compared to cells treated with a control siRNA (siCONT). (H) Quantification of cilia length shown in (G). Cilia length in A204 cells transfected with siKif7 was significantly increased compared to siCONT. n = 150. Error bars represent SD. p < 0.002, for an unpaired t test. (I) Western blot showing Kif7 levels in A204 cells transfected with control siRNA or Kif7 siRNA (indicated) for experiments shown in (F)–(H).

Next, we wanted to understand the molecular nature of the longer cilia phenotype and whether cilia length could be responsible for the observed changes in drug response. The kinesin Kif7 has been shown to control cilia length by organizing the cilia tip in coordination with the IFT-B particle IFT81 (He et al., 2014). The changes in cilia length observed in DasR cells were reminiscent of those seen in Kif7-deficient cells, suggesting that Kif7 could be involved in this phenotype. In parental A204 cells, we found that Kif7 localized to the ciliary base, along the cilium, and at the cilium tip, as previously described (He et al., 2014) (Figure 2A). In contrast, A204 DasR cells had a significant decrease in Kif7 localization to the axoneme and cilia tip (Figure 2A), while total Kif7 levels remained unchanged (Figure 2E). We also found that, in control A204 cells, IFT81 localized along the axoneme and at the cilia tip. However, this was significantly reduced in DasR cells (Figure 2B). The microtubule plus-end-binding protein EB1, which localizes to centrioles and cilia tips (Pedersen et al., 2003), is also thought to play a role in cilia biogenesis (Schrøder et al., 2011). We found that the localization of EB1 was restricted to centrioles in control A204 cells, while in DasR cells EB1 localized along the ciliary axoneme as well as the cilia tip (Figures 2C and 2D). Thus, control of cilia length and cilia tip compartment organization, as well as cilia transport appear compromised in DasR cells. For the other models presented in this study, the cilia length increase did not correlate with defects in Kif7 localization (not shown). This is likely due to differences in cellular and genetic context across models, and argues that diverse molecular pathways might drive resistance through their effects on ciliogenesis and/or cilia length.

Kif7 inactivation has been shown to destabilize cilia (He et al., 2014). Thus, we tested the stability of cilia in DasR cells by subjecting them to cold treatment or the microtubule-destabilizing agent nocodazole. Both treatments initially resulted in a significantly increased rate of cilia shortening in DasR cells compared to parental A204. However, by 60 min (30 min for nocodazole), this difference in cilia length was no longer evident (Figures S3D–S3G).

We reasoned that if cilia length was involved in drug resistance, targeting cilia length control could modulate drug response. Since Kif7 inactivation has been shown to increase cilia length, we first downregulated Kif7 in parental A204 cells and assessed their response to dasatinib. Notably, Kif7-depleted cells continued to grow in the presence of Dasatinib, while control-transfected cells were growth arrested by the inhibitor (Figure 2F). We observed a modest level of non-specific transfection-induced toxicity in A204 cells. However, the overall cell cycle profile of these cells was unaffected (Table S1). Kif7 depletion also caused the expected increase in cilia length (Figures 2G–2I). Thus, dasatinib resistance in these cells seems to involve misregulation of cilia length.

### Kinase Inhibitor-Resistant Cells Show Increased Hedgehog Pathway Activation

**Figure 3:**
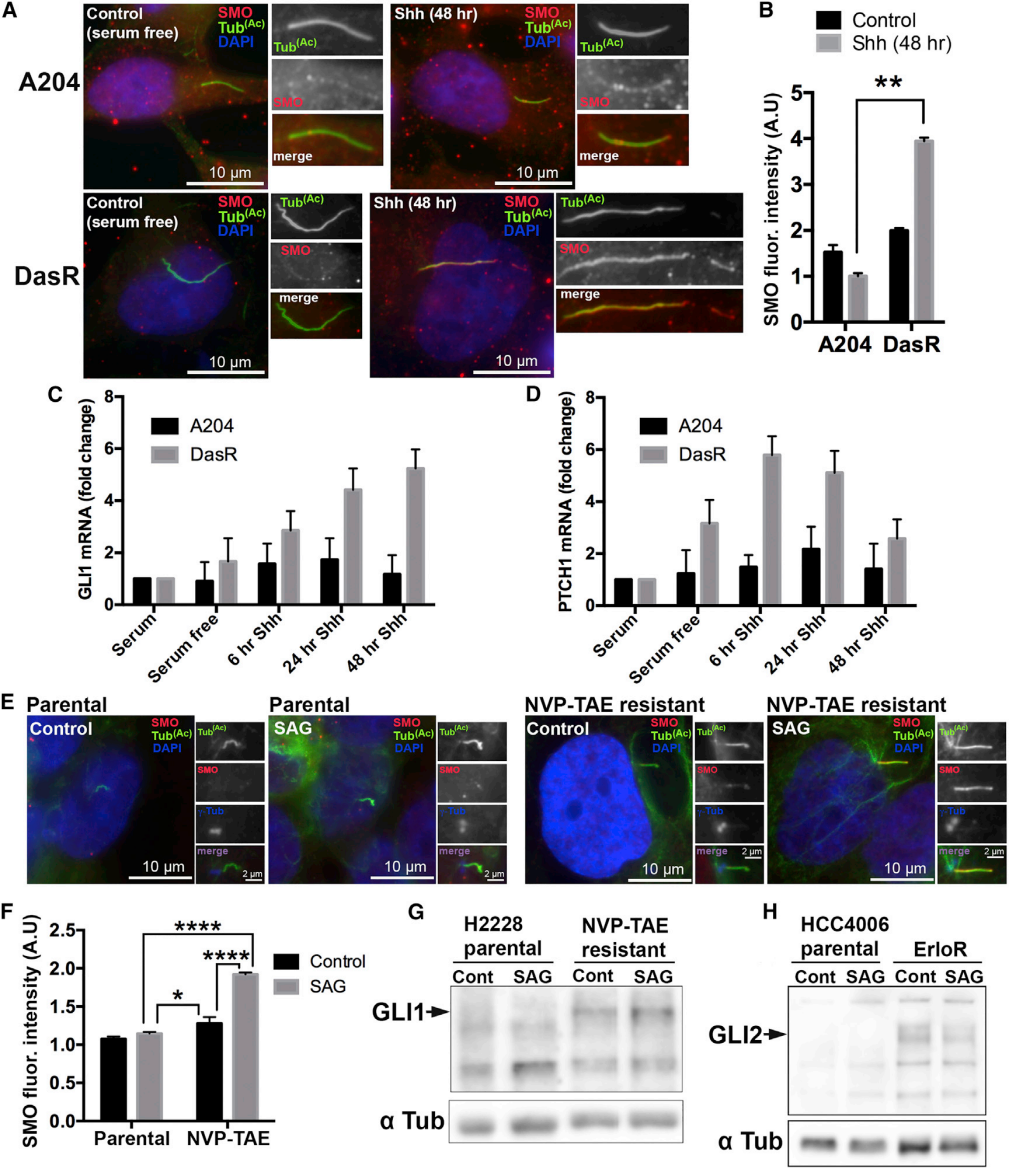
Kinase Inhibitor-Resistant Cells Show Increased Hedgehog Pathway Activation (A) A204 cells (top panels) or a dasatinib-resistant subline (DasR) (bottom panels) were serum starved for 24 hr, and then either left untreated (left) or treated with human Sonic Hedgehog (SHH) (5 μg/mL) (right) for an additional 48 hr. Cells were then fixed and stained for acetylated tubulin to mark cilia (green), Smoothened (SMO) (red), and with DAPI (blue). DasR cells show increased SMO localization to cilia compared to control A204 cells. (B) Quantification of SMO cilia fluorescence intensities for the experiment shown in (A). Fluorescence intensity was normalized to surrounding fluorescence. n = 150. Error bars represent SD. p < 0.01, for an unpaired t test. (C and D) qPCR showing mRNA levels of Hh target genes of *GLI1* (C) and *PTCH1* (D) in A204 and DasR cells before serum starvation, after 24 hr of serum starvation and after stimulation with 5 μg/mL SHH for the times indicated. TATA box-binding protein (*TBP*) was used as a reference gene, and fold change was calculated by comparing mRNA levels relative to control (serum). (E) NCI-H2228 parental or a NVP-TAE684 -resistant subline (NVP-TAE) was serum starved for 24 hr, and then either left in serum-free media or treated with human SAG (100 nM) for an additional 48 hr. Cells were then fixed and stained for acetylated tubulin to mark cilia (green), Smoothened (SMO) (red), and with DAPI (blue). NVP-TAE684-resistant cells show increased SMO localization to cilia compared to parental cells. (F) Quantification of SMO cilia fluorescence intensities for the experiment shown in (E). Fluorescence intensity was normalized to surrounding fluorescence. n = 150. Error bars represent SD. p < 0.0001 (parental SAG versus NVP-TAE684 SAG), p < 0.004 (parental SAG versus NVP-TAE684 control), and p < 0.0001 (NVP-TAE684 control versus NVP-TAE684 SAG), for Tukey’s multiple-comparison test. (G) Western blot showing GLI1 levels in NCI-H2228 parental cells and the NVP-TAE684-resistant subline. Cells were serum starved for 24 hr, and then either left in serum-free media or treated with human SAG (100 nM) for an additional 48 hr. Note the increased expression of Gli1 in NVP-TAE684-resistant cells compared to parental cells. (H) Western blot showing Gli2 levels in HCC4006 parental cells and the ErloR-resistant subline. Cells were serum starved for 24 hr, and then either left in serum-free media or treated with human SAG (100 nM) for an additional 48 hr. Note the increased levels of Gli2 in ErloR-resistant cells compared to parental cells.

Because drug resistance often involves aberrant activation of compensatory pathways (many of which reside in or are controlled by cilia), we hypothesized that the observed changes in cilia would lead to misregulated cilia-dependent signaling. Activation of the evolutionarily conserved Hedgehog (Hh) pathway requires a functional cilium, and it is coordinately regulated at the body of the cilium and the cilium tip (Goetz et al., 2009, Huangfu and Anderson, 2005). Given that changes in cilia tip organization, KIF7 defects, and changes in ciliogenesis have been shown to disrupt the Hedgehog pathway (He et al., 2014), we hypothesized that cilia changes in resistant cells might affect Hh function. We first examined SMO recruitment to the cilium following SHH pathway stimulation (Figure 3A). Fluorescence intensity quantification showed increased SMO recruitment to cilia in DasR cells compared to control cells (Figures 3A, 3B, S4A, and S4B). To assess the functional relevance of this increase, we examined transcriptional targets of Hh activation by RT-PCR at steady state and at different time points after addition of human SHH. We found significantly higher induction of the Hh target genes GLI1 and PTCH1 in response to either SHH (Figures 3C and 3D) or the SMO agonist (SAG) (Figures S4C and S4D) in DasR cells compared to A204 control cells, thereby confirming that the longer cilia observed in DasR cells support enhanced Hh pathway activation (Figures 3A–3D and S4A–S4D). Consistently, lung cancer H2228 cells with acquired resistance to the ALK inhibitor NVP-TAE684 also showed increased Hedgehog pathway activation (seen as both a significant increase in ciliary localization of SMO following receptor engagement [Figures 3E and 3F], and an increase in the levels of GLI1 ([Figure 3G]) compared to parental cells. Additionally, we observed an increase in GLI2 levels in erlotinib-resistant HCC4006 cells compared to parental controls (Figure 3H).

### Cilia and Ciliary Pathways Are Important Mediators of Kinase Inhibitor Resistance

**Figure 4:**
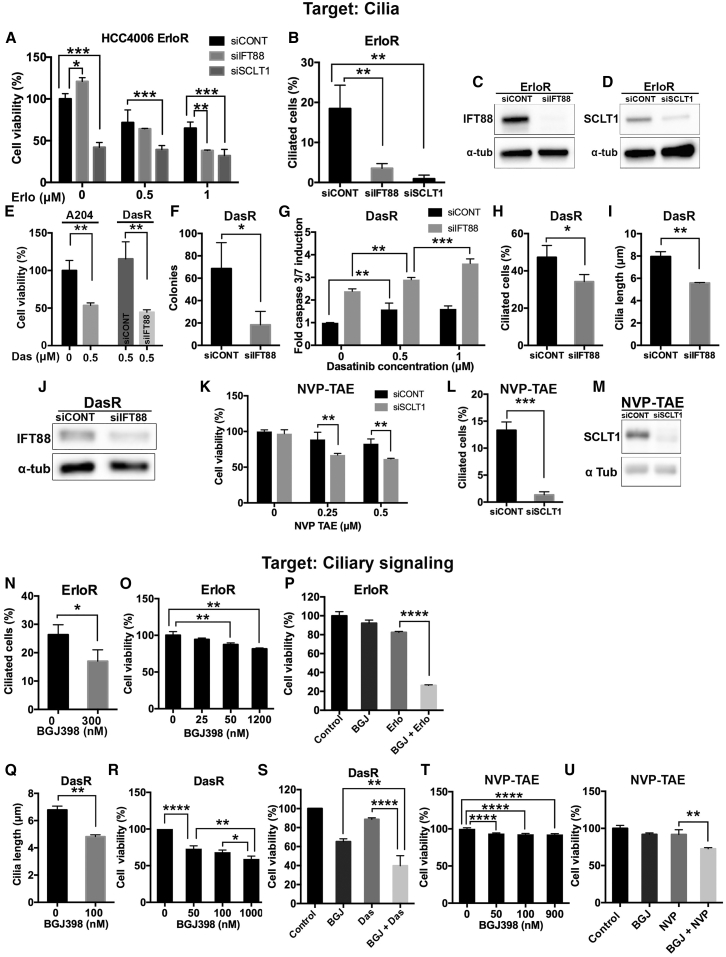
Cilia and Ciliary Pathways Are Important Mediators of Kinase Inhibitor Resistance (A) Cell viability (CellTiter-Glo) in HCC4006 cells grown in erlotinib (indicated) were transfected with either control siRNA, IFT88 siRNA, or SCLT1 siRNA (indicated). Cell viability was normalized to siCONT DMSO control (0 μM)-treated cells (n = 3). Error bars represent SD. p < 0.05 (siControl compared to siIFT88 at 0 μM), p < 0.0001 (siControl versus siSCLT1 at 0 μM), p < 0.0008 (siControl versus siSCLT1 at 0.5 μM), p < 0.006 (siControl compared to siIFT88 at 1 μM), and p < 0.0007 (siControl versus siSCLT1 at 1 μM), Tukey’s multiple-comparison test. (B) Cilia quantification in HCC4006-erlotinib-resistant subline (ErloR) transfected with either control siRNA, siRNA for IFT88 (siIFT88) (Smartpool), or SCLT1 siRNA (siSCLT1) (Smartpool). Note that, in both cases, cilia frequency is significantly decreased compared to control siRNA. n = 300. Error bars represent SD. p < 0.003 (siCONT versus siIFT88) and p < 0.005 (siCONT versus siSCLT1). (C and D) Western blots showing IFT88 (C) or SCLT1 (D) levels in ErloR cells, transfected with control siRNA and either IFT88 siRNA (C) or SCLT1 siRNA (D) for the experiments shown in (A) and (B). (E) Cell viability in A204 cells, grown in the absence or the presence of dasatinib (indicated), and in DasR cells, after transfection with control siRNA or IFT88 siRNA (Robert et al., 2007). Cell viability is normalized to A204 DMSO control (0 μM) (n = 3). Error bars represent SD. p < 0.005 for A204 grown in 0 dasatinib compared to 0.5 μM dasatinib and p < 0.006 for DasR siCONT compared to siIFT88, unpaired t test. (F) Soft agar colony formation in DasR cells transfected with control siRNA or siRNA for IFT88 (Robert et al., 2007). Error bar bars represent SD. p < 0.03, unpaired t test; n = 3. (G) Caspase 3/7 activity of DasR cells after treatment with dasatinib in control cells (siCONT) or upon downregulation of IFT88 (siIFT88). Fold change in caspase 3/7 activity was normalized to siCONT (DMSO), n = 3, p < 0.003 (siCONT 0 μM versus siCONT, 0.5 μM), < 0.002 (siIFT88 0 μM versus siIFT88, 0.5 μM), p < 0.006 (siIFT88, 0.5 μM, versus siIFT88, 1 μM), < 0.002 (siCONT, 0 μM, versus siCONT, 1 μM), and < 0.0001 (siIFT88, 0 μM, versus siIFT88, 1 μM), Tukey’s multiple-comparison test. (H and I) Quantification of percent ciliated cells (n = 300) (H) and cilia length (n = 150) (I) for DasR cells transfected with an IFT88 siRNA (siIFT88) (Robert et al., 2007) or control siRNA (siCONT). Error bars represent SD. p < 0.04 for (H) and p < 0.0008 for (I), unpaired t test. (J) Western blot showing IFT88 levels in DasR cells transfected with control siRNA or IFT88 siRNA (indicated) for experiments shown in (E)–(I). (K) Cell viability in NCI-H2228 NVP-TAE684-resistant cells (NVP-TAE) after treatment with NVP-TAE684 in control cells (siCONT) or upon downregulation of SCLT1 (siSCLT1). Cell viability was normalized to siCONT (DMSO). n = 3, p < 0.006 for 0.25 μM, and p < 0.006 for 0.5 μM, unpaired t test. Note that, in the absence of cilia, NVP-TAE684-resistant cells become more sensitive to the inhibitor. (L) Quantification of percent ciliated cells (n = 300) for the experiment shown in (K). Error bars represent SD. p < 0.0003, unpaired t test. (M) Western blot showing SCLT1 levels in NCI-H2228 NVP-TAE684-resistant cells transfected with siCONT or siSCLT1 (indicated) for the experiments shown in (K) and (L). (N) Quantification of ciliated cells (percent) for HCC40006 erlotinib-resistant cells (ErloR) treated with or without the FGFR inhibitor BGJ398 for 72 hr. Note that, after treatment with BGJ398, cilia length was reduced. n = 150. Error bars represent SD. p < 0.04, unpaired t test. (O) Cell viability (CellTiter-Glo) of the HCC4006 erlotinib-resistant subline (ErloR) grown in a range of concentrations for the FGFR inhibitor BGJ398. Cell viability was normalized to DMSO control (n = 3). Error bars represent SD. p < 0.004 (0 compared to 50 nM, BGJ398) and p < 0.0004 (0 compared to 1,200 nM BGJ398), Tukey’s multiple-comparison test. (P) Cell viability (CellTiter-Glo) of ErloR grown in 1 μM erlotinib (Erlo), 300 nM BGJ398, or both. Note that combining both erlotinib and BGJ398 significantly reduced growth compared to erlotinib used as a single agent. Cell viability was normalized to DMSO control (n = 3). Error bars represent SD. p < 0.0001, for an unpaired t test. (Q) Cilia length of A204 dasatinib-resistant cells (DasR) treated with or without BGJ398 for 24 hr in reduced serum conditions (5% FBS). Note that, after treatment with BGJ398, cilia length in DasR cells was reduced. n = 150. Error bars represent SD. p < 0.0005, unpaired t test. (R) Cell viability (CellTiter-Glo) of the A204 Das-resistant subline (DasR) grown in a range of BGJ398 concentrations. Cell viability was normalized to DMSO control (n = 3). Error bars represent SD. p < 0.0001 (0 compared to 50 nM, BGJ398), p < 0.003 (50 nM compared to 1,000 nM), and p < 0.03 (100 nM compared to 1,000 nM), Tukey’s multiple-comparison test. (S) Cell viability (CellTiter-Glo) of DasR cells treated with dasatinib (0.5 μM), the FGFR1 inhibitor BGJ398 (100 nM), or a combination of both. n = 3. Cell viability is normalized to the DMSO control. Error bars represent SD. p < 0.004 (BGJ versus BGJ + Das) and p < 0.0001 (Das versus BGJ + Das), Tukey’s multiple-comparison test. (T) Cell viability (CellTiter-Glo) of the H2228 NVP-TAE684-resistant subline (NVP-TAE684) grown in a range of BGJ398 concentrations. Cell viability was normalized to DMSO control (n = 3). Error bars represent SD. p < 0.0001 (0 compared to 50, 100, and 900 nM, BGJ398), Tukey’s multiple-comparison test. (U) Cell viability (CellTiter-Glo) of NCI-H2228 NVP-TAE684-resistant cells treated with NVP-TAE684 (0.5 μM), BGJ398 (1.2 μM), or both. Cell viability is normalized to the DMSO control. Error bars represent SD. p < 0.002, for an unpaired t test.

Our results indicate that acquired resistance to kinase inhibitors is associated with the upregulation of a number of ciliogenesis pathways, and suggest that targeting cilia might be an effective strategy to overcome resistance. To test this hypothesis, we asked whether inhibition of ciliogenesis via knockdown of the centriole distal appendage protein SCLT1 (Tanos et al., 2013) or the IFT-B particle IFT88 (Pazour et al., 2000) could affect KIR cell viability. While disrupting ciliogenesis in our three models of acquired drug resistance had negligible effects in cell cycle distribution (Table S1), it sensitized drug-resistant cells to the appropriate kinase inhibitor (i.e., erlotinib, dasatinib, or NVP-TAE684) (Figures 4A, 4E, and 4K). Furthermore, IFT88 knockdown in DasR cells significantly reduced anchorage-independent growth (Figure 4F) and increased apoptosis in the presence of the inhibitor (Figure 4G).

Drug resistance has been associated with entry of tumor cells into a quiescent state (Sharma et al., 2010, Yeh and Ramaswamy, 2015). However, we did not observe any changes in the G0/G1 fraction in any of the models we studied (Table S1).

Next, we interrogated the impact of pharmacological targeting of cilia function on drug resistance. First, we focused on the Hh pathway because it is upregulated in DasR cells and because it has previously been implicated in drug resistance (Faião-Flores et al., 2017). Interestingly, we found that treatment with GANT61, a small-molecule inhibitor of the Hh pathway (Lauth et al., 2007), reduced viability in both DasR and control cells (Figure S4E), highlighting the overall importance of the Hedgehog pathway in certain cancers. Notably, treatment with the Gli inhibitor GANT61 or the Smoothened inhibitor vismodegib significantly sensitized resistant cells to the relevant kinase inhibitor (Figures S4G, S4H, and S4J). Second, we targeted the FGFR because it has been previously shown to control ciliogenesis and cilia length (Neugebauer et al., 2009). We found that treatment of erlotinib-resistant HCC4006 cells with the specific FGFR inhibitor BGJ398 significantly reduced cilia formation (Figure 4N), and more importantly, it re-sensitized these cells to erlotinib (Figure 4P). We found similar results when we evaluated FGFR inhibition in A204 DasR cells (Figures 4Q–4S) and NVP-TAE684-resistant NCI-H2228 cells (Figures 4T–4U), suggesting that inhibition of cilia regulators such as FGFR may represent a good therapeutic strategy to overcome drug resistance in a variety of contexts.

### *De Novo* Drug Resistance Is Also Associated with Increased Ciliogenesis

**Figure 5:**
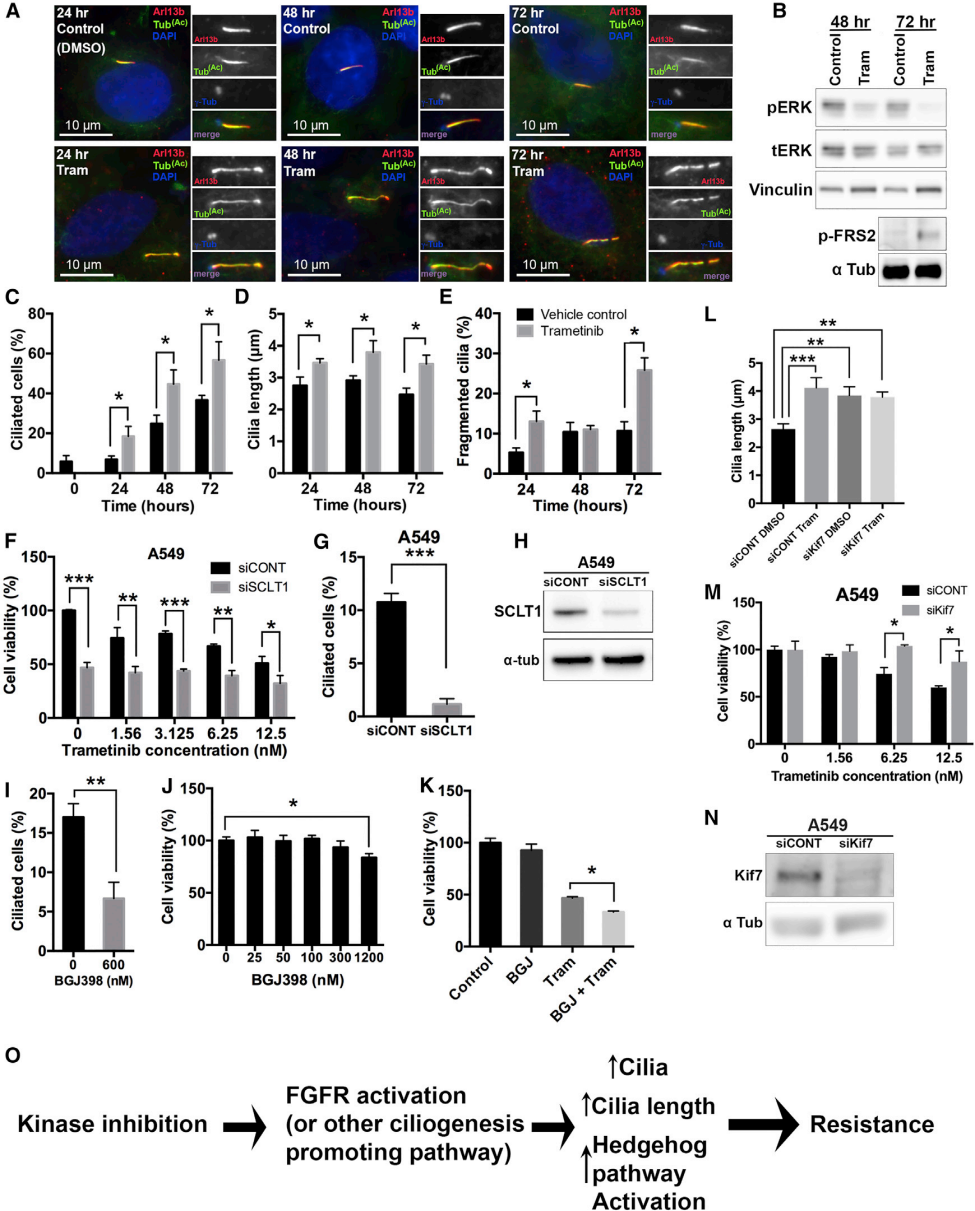
*De Novo* Drug Resistance Is Associated with Increased Ciliogenesis and Ciliary Signaling (A) A549 cells were treated with 50 nmol/L trametinib (Tram) or DMSO (vehicle control) for the indicated times, and then fixed and stained with antibodies for acetylated tubulin (green), Arl13B (red), γ-tubulin (blue/inset), and DAPI (blue). Note that exposure to trametinib promoted a significant increase in cilia, cilia length, and fragmentation. (B) Western blot showing levels of phosphorylated ERK, total ERK, vinculin (loading control), phospho-FRS2, and α-tubulin (loading control) in the presence and absence of 50 nM trametinib (Tram) in A549 cells. Note that, after 48 and 72 hr of trametinib exposure, pERK was reduced. Phospho-FRS2 levels increased after 72 hr of trametinib treatment. (C–E) Quantification of ciliated cells (C), cilia length (D), and fragmentation (E) for cells shown in (A). n = 150 cilia. Error bars represent the SD. p < 0.05 unpaired t test. (F) Cell viability in A549 cells after treatment with the MEK inhibitor trametinib in control cells (siCONT) or upon downregulation of the distal appendage protein SCLT1 (siSCLT1). Cell viability was normalized to siCONT DMSO control (0 nM) cells. n = 3, p < 0.0001 for 0 nM, p < 0.008 for 1.56 nM, p < 0.0009 for 6.25 nM, and p < 0.003 for 12.5 nM, unpaired t test. (G) A549 cells transfected with siRNA for SCLT1 (siSCLT1) had reduced ciliated cells compared to siRNA control (siCONT). n = 300. Error bars represent SD. p < 0.0001. (H) Western blot showing SCLT1 levels in A549 cells transfected with control siRNA or SCLT1 siRNA (indicated) for the experiments shown in (F) and (G). (I) Cilia quantification of A549 cells treated with or without the FGFR inhibitor BGJ398 for 48 hr. Note that, after treatment with BGJ398, the percentage of ciliated cells was reduced. n = 150. Error bars represent SD. p < 0.003, unpaired t test. (J) Cell viability (CellTiter-Glo) of A549 cells grown in a range of BGJ398 concentrations. Cell viability was normalized to DMSO control (n = 3). Error bars represent SD. p < 0.02, Tukey’s multiple-comparison test. (K) Cell viability (CellTiter-Glo) of A549 cells treated with trametinib (6.25 nM), BGJ398 (300 nM), or a combination of both. n = 3. Cell viability is normalized to the DMSO control. Error bars represent SD. p < 0.02, for an unpaired t test. (L) Quantification of cilia length (n = 150) of A549 cells transfected with control siRNA (siCONT) or Kif7 siRNA (siKif7) and treated with either DMSO or trametinib (50 nM) for 48 hr. Error bars represent SD. p < 0.0009 (siCONT DMSO versus siCONT Tram), p < 0.004 (siCONT DMSO versus siKif7 DMSO), and p < 0.005 (siCONT DMSO versus siKif7 Tram), Tukey’s multiple-comparison test. (M) Cell viability in A549 cells after treatment with trametinib in control cells (siCONT) or upon downregulation of Kif7 (siKif7). Cell viability was normalized to 0 nM for both siCONT and siKif7. n = 4. p < 0.0006 for 6.25 nM and p < 0.01 for 12.5 nM, unpaired t test. (N) Western blot showing Kif7 levels in A549 cells transfected with control siRNA (siCONT) or Kif7 siRNA (siKif7) for the experiment shown in (L) and (M). (O) Proposed model for upregulation of ciliogenesis leading to kinase inhibitor resistance.

Finally, we wanted to know whether cilia changes were associated with any instances of *de novo* drug resistance. To address this question, we used a previously described model of *de novo* drug resistance in K-Ras mutant cells (Kitai et al., 2016, Manchado et al., 2016). Because direct targeting of Ras has been challenging, inhibiting components of the downstream MAPK pathway, including mitogen-activated protein kinase kinase (MEK) has been pursued as an alternative strategy. However, KRAS mutant cells are largely refractory to these drugs. Two independent studies found that, in KRAS mutant lung cancer cells, FGFR can mediate adaptive resistance to the MEK inhibitor trametinib (Kitai et al., 2016, Manchado et al., 2016). We therefore hypothesized that MEK-inhibitor resistance in KRAS mutant A549 cells would be accompanied by changes in ciliogenesis. We found that cilia number as well as cilia length were upregulated in A549 cells following trametinib treatment (Figures 5A–5E), although changes in Hedgehog pathway activation were difficult to assess due to the significantly high level of basal activity (Figures S5J–S5K). Additionally, KRAS mutant NCI-H1792 and NCI-H23 lung cancer cells also showed upregulated ciliogenesis and increased Hedgehog pathway activation (Figures S5A–S5I and S5K; Table 1) in response to MEK inhibition. Furthermore, after 24 hr of drug treatment, the elongated cilia in A549 cells started to show evidence of fragmentation (Figures 5A and 5E). Thus, these results suggest that release of terminal cilia fragments might be a common feature of KIR cells independently of the molecular identity of the resistance pathway.

Importantly, inhibiting ciliogenesis in all three KRAS mutant lines reduced their viability when combined with trametinib (Figures 5F, S6A, and S6D), while having no significant changes in cell cycle distribution on its own (Table S1). Furthermore, pharmacological suppression of ciliogenesis via treatment with the FGFR inhibitor BGJ398 significantly reduced the viability of these cells in the presence of trametinib (Figures 5K, S6K, and S6O), providing a potential mechanism for the previously described synergistic effects of combined MEK and FGFR inhibitor treatment in these cells (Kitai et al., 2016, Manchado et al., 2016). In contrast, promoting cilia lengthening via Kif7 knockdown (Figures 5L and 5N) significantly right-shifted the response to trametinib in A549 cells (Figure 5M). These data strongly suggest that similar to our models of acquired kinase inhibitor resistance, changes in ciliogenesis can also mediate *de novo* adaptive resistance.

These data support a model wherein inhibition of certain kinases leads to increased activation of FGFR (or other cilia promoting pathways), leading to enhanced ciliogenesis and concomitant Hedgehog pathway activation, thus facilitating the generation of inhibitor insensitive survival signals (Figure 5O). Cilia could thus function as a permissive platform for a number of drug resistance mechanisms with broad therapeutic implications.

## Discussion

Our work suggests that ciliogenesis and cilia function as key biological processes that play permissive roles in the emergence of resistance to kinase inhibitors in cancer cells. Cilia have been shown to have opposing roles in tumorigenesis, depending on the nature of the driver oncogenic lesion (Han et al., 2009, Wong et al., 2009). Our data show that resistance to a variety of targeted therapies in several experimental models is characterized by an increase in the number and/or length of primary cilia and by cilia fragmentation. The latter is associated with decreased cilia polyglutamylation, a modification known to destabilize microtubules, which could contribute to cilia fragmentation. It is not clear how changes in the polyglutamylated fraction of cilia would influence drug response. However, it has been shown that changes in polyglutamylation can bias the recruitment of specific proteins to α-tubulin through its effects on the binding affinity of select motor proteins (Ikegami et al., 2007), which could potentially affect cilia-directed survival signaling.

Consistent with the notion that aberrant cilia can alter oncogenic signaling, we find that the local abundance of a number of cilia-associated oncoproteins changes in resistant cells. For example, DasR cells lose PDGFRα expression (Figure S1F) (Wong et al., 2016), show a slight increase in FGFR1 (Figure S1G), and have increased ciliary localization of IGF-1R (Figures S1H–S1I).

We find that defects in cilia length control are involved in the regulation of cilia-dependent drug resistance. One mechanism of cilia elongation leading to drug resistance involves a decrease in Kif7/IFT81 localization to cilia. In parental A204 cells, we find that Kif7 is at the cilia tip, where it promotes microtubule plus-end catastrophe, thus creating a tip compartment for the enrichment of IFT81 (He et al., 2014). In contrast, in DasR cells, the Kif7-rich cilia tip compartment is lost, which explains the absence of IFT81. Additionally, we observed increased EB1 localization along the cilia and cilia tips in KIR cells (Figures 2C and 2D), which is suggestive of defective diffusion barrier control. Thus, KIR cells have clearly defined molecular changes at the cilia tips. These data raised the question of whether aberrant cilia lengthening might be sufficient to confer drug resistance. Notably, increasing cilia length through downregulation of Kif7 in either A204 or A549 cells did in fact promote resistance to dasatinib and trametinib, respectively (Figures 2F and 5M).

Our results show that KIR cells have an enhanced response to Hedgehog pathway activation (Figures 3, S4, and S5). Interestingly, treatment of resistant cells with the Gli-selective inhibitor GANT61 or the clinical SMO inhibitor vismodegib re-sensitized these cells to the relevant kinase inhibitor (Figures S4G, S4H, S4J, S5L, and S5M), suggesting that drug resistance may be mediated by a critical effector of cilia-dependent signaling.

Interestingly, we and others have shown that resistance to both the MEK inhibitor trametinib and the tyrosine kinase inhibitor dasatinib can be mediated by activation of FGFR (Manchado et al., 2016, Kitai et al., 2016, Wong et al., 2016) (Figure 5B), a kinase known to regulate cilia length (Neugebauer et al., 2009). Notably, we found that treatment of KIR cells with an FGFR inhibitor not only restored kinase inhibitor sensitivity (Figures 4P, 4S, 4U, 5K, and S6) but also reduced cilia and/or cilia length (Figures 4N, 4Q, 5I, and S6). Similarly, targeting ciliogenesis through knockdown of the centriole distal appendage protein SCLT1 (Tanos et al., 2013) or the IFT particle IFT88 (Pazour et al., 2000) sensitized cells to the relevant kinase inhibitor (Figures 4A, 4E, 4K, 5F, and S6).

This is in contrast to the lack of sensitizing activity of therapeutic agents that cause growth arrest in specific phases of the cell cycle (e.g., cisplatin, rapamycin, or doxorubicin), which suggests that the sensitizing effects of ciliogenesis inhibition in drug-resistant cells are unlikely to be attributed to cell cycle deregulation (Figure S7). It is also unlikely that drug resistance-associated changes in cilia are caused by alterations in cell cycle-dependent signals (Plotnikova et al., 2009), given that drug-resistant cells did not show any significant differences in cell cycle distribution compared to their parental counterparts (Table S1).

Because cilia can extend during G0, and quiescence has been associated with drug resistance, we examined the kinetics of cell cycle re-entry in our isogenic models following serum starvation and re-challenge. However, we did not find any correlation between time to re-entry and the observed changes in cilia, suggesting quiescence is unlikely to be the cause of aberrant ciliation or drug resistance in our models (Table S2).

Of note, we have also examined ciliation in an A549 isogenic model of acquired chemoresistance and found that resistance to cisplatin and vinflunine is also associated with a significant increase in ciliogenesis (Figures S2M–S2O; Table 1), suggesting that cilia might be involved in resistance to a wide range of therapeutic agents.

In summary, our study shows that aberrant ciliogenesis could serve as a functional platform for a variety of cancer drug resistance mechanisms (both *de novo* and acquired) and provides rationale for a broad therapeutic strategy to overcome resistance in a variety of settings.

## Experimental Procedures

### Cell Culture

Cells were maintained in DMEM (A549, A204, and the dasatinib-resistant subline DasR), and DME/F12 (HCC4006) containing 10% fetal bovine serum (FBS), 4 mM GlutaMax (Thermo Scientific, Waltham, MA), 500 μg/mL Normocin (InvivoGen, San Diego, CA), 100 units/mL penicillin, and 100 mg/mL streptomycin (Thermo Scientific). 5 μM dasatinib (LC Labs, Woburn, MA) was supplemented to DasR growth media. The erlotinib-resistant HCC4006 subline was grown in the presence of 1 μM erlotinib (LC Labs). NCI-H23 and NCI-H1792 were maintained in RPMI containing 10% FBS, 2 mM GlutaMax (Thermo Scientific), 500 μg/mL Normocin (InvivoGen), 100 units/mL penicillin, and 100 mg/mL streptomycin (Thermo Scientific). NCI-H2228, PC9, A549, and the cisplatin, carboplatin, and vinflunine-resistant sublines were maintained in Iscove’s modified Dulbecco’s medium (IMDM) containing 10% FBS, 2 mM GlutaMax (Thermo Scientific), 500 μg/mL Normocin (InvivoGen), 100 units/mL penicillin, and 100 mg/mL streptomycin (Thermo Scientific). The NVP-TAE684-resistant NCI-H2228 subline was supplemented with 0.5 μM NVP-TAE684 (Axon Medchem; catalog #Axon 1416). PC9-resistant sublines were cultured with 2 μM afatinib (Stratech Scientific; catalog #S1011-SEL) and 10 μM erlotinib (LC Labs). A549-chemoresistant sublines were cultured in 2 μg/mL cisplatin (Cayman Chemical Company, Ann Arbor, MI) or 10 μg/mL carboplatin (Cayman Chemical Company). HEK293T cells were maintained with DMEM containing 10% FBS, 2 mM GlutaMax (Thermo Scientific), 100 units/mL penicillin, and 100 mg/mL streptomycin (Thermo Scientific).

### Ciliogenesis Experiments

To induce cilia formation, cells were plated on to poly-lysine-coated coverslips in 3.5-cm plates at 0.4 × 10^6^ cells per well, allowed to attach for 24 hr, and then serum starved for 48 hr. For A549, NCI-H23, and NCI-H1792, ciliogenesis experiments were carried out in the presence of serum, since trametinib proved to be toxic otherwise. To activate the Hedgehog pathway, cells were serum starved for 24 hr prior to the addition of 5 μg/mL SHH-N (Peprotech, London, UK) or 100 nM SAG (Millipore, Darmstadt, Germany). For cilia stability experiments, cells were either incubated in 4°C culture media or treated with 10 μM nocodazole (Sigma-Aldrich, St. Louis, MO).

### Immunofluorescence

Cells were fixed in 4% paraformaldehyde for 10 min at room temperature, for the following antibodies: mouse anti-α-tubulin (1:200; YL1/2; Bio-Rad; MCA77G), mouse anti-acetylated tubulin (1:2,000; 6-11B-1; Sigma; T7451), rabbit anti-Arl13B (1:500; Proteintech; 17711-1-AP), mouse anti-EB1 (1:250; BD Biosciences; 5/EB1), mouse anti-centrin (1:500; 3E6; Abnova; H00001070-M01), rabbit anti-detyrosinated α-tubulin (1:100; Abcam; ab48389), rabbit anti-IGF-1Rβ (1:250; C-20; Santa Cruz; sc-713), mouse anti-polyglutamylated tubulin (1:200; GT335; Adipogen; AG-20B-0020), and rabbit anti-SMO (a kind gift from Kathryn Anderson; 1:500). An additional fixation step of 20 min in cold methanol was used for rabbit anti-IFT88 (1:500; Proteintech; 13967-1-AP) and mouse anti-γ-tubulin (1:500; TU-30; Santa Cruz; sc-51715). For antibodies against Kif7 (1:500; rabbit polyclonal; kind gift from Kathryn Anderson’s lab) and rabbit anti-IFT81 (1:200; Proteintech; 11744-1-AP), cells were first permeabilized for 2 min in PTEM buffer (20 mM PIPES [pH 6.8], 0.2% Triton X-100, 10 mM EGTA, and 1 mM MgCl_2_) followed by fixation in cold methanol for 20 min. After fixation, cells were permeabilized for 5 min in 0.1% Triton X-100 in PBS, then blocked with 3% (w/v) bovine serum albumin in PBS and 0.1% Triton X-100 for 5 min. Primary antibodies were diluted in blocking solution and incubated for 1 hr followed by three washes with PBS and 0.1% Triton X-100. After that, goat secondary antibodies conjugated to either Alexa Fluor 488, 594, or 680 (1:500 dilution; Thermo Scientific) were incubated for 1 hr followed by three washes and incubation with DAPI (Thermo Scientific).

### Image Acquisition and Analysis

Fluorescent images were acquired on an upright microscope (Axio Imager M2; Zeiss) equipped with 100× oil objectives, 1.4 numerical aperture (N.A.), a camera (ORCA R2; Hamamatsu Photonics), and a computer with image-processing software (Zen). Images were quantified for pixel density and cilia length using ImageJ and MATLAB and assembled into figures using Photoshop (CS5; Adobe). For pixel density quantification, images were taken using equal settings. ImageJ pixel density quantification measured the mean gray value (sum of the gray values of all the pixels in the selection divided by the number of pixels), of cilia defined by either Arl13b or acetylated tubulin staining.

For MATLAB quantifications, we used a custom-written script to quantify fluorescent intensity profiles along cilia. Cilia were segmented in a user-interactive manner using the improfile function from MATLAB. Improfile retrieves the intensity values of pixels along a multiline path defined by the user. Acetylated tubulin or Arl13B was used to define the cilium path, and intensity profiles were retrieved from channels of interest. To reduce noise, we measured the average fluorescent intensity of 3 pixels (above, on, and below the path) for each position along the cilium. To compare intensity profiles along cilia of different lengths, we divided cilium length into ten bins and extracted the average fluorescent intensity for each bin. The script is available upon request. 3D structured illumination images were acquired using an SR1 Elyra PS1 microscope (Zeiss), images were processed using the ImageJ plugin SIMcheck to remove artifacts, and 3D videos were made using the Volocity software (PerkinElmer).

### Western Blots

Cells were lysed in RIPA buffer (Sigma-Aldrich) supplemented with protease and phosphatase inhibitors (Thermo Scientific) on ice. Lysates were sonicated and cleared by centrifugation at 12,000 × *g* at 4°C for 30 min. Samples were separated by SDS-PAGE on 3–8% polyacrylamide gradient gels followed by transfer to nitrocellulose membranes. Membranes were probed with primary antibodies against mouse anti-Erk (1:1,000; 3A7; Cell Signaling; 9107), rabbit anti-phospho-Erk (1:1,000; Cell Signaling; 9101), rabbit anti-Met (1:1,000; D1C2; Cell Signaling; 8198), rabbit anti-phospho-Met (1:1,000; D26; Cell Signaling; 3077), rabbit anti-EGFR (1:1,000; D38B1; Cell Signaling; 4267), rabbit anti-phospho-EGFR (1:1,000; D7A5; Cell Signaling; 3777), rabbit anti-Gab1 (1:1,000; Cell Signaling; 3232), rabbit anti-phospho-Gab1 (1:1,000; C32H2; Cell Signaling; 3233), mouse anti-GLI1 (1:750; L42B10; Cell Signaling; 2643), rabbit anti-phospho-FRS2-α (1:500; Cell Signaling; 3864), rabbit anti-GLI2 (1:500; H-300; Santa Cruz; sc-28674), mouse anti-FLAG (1:1,000; M2; Sigma; F1804), rabbit anti-IGF-1Rβ (1:250; C-20; Santa Cruz; sc-713), rabbit anti-Kif7 (1:500), rabbit anti-SCLT1 (1:500; Sigma; HPA036560), mouse anti-β-Actin (1:2,000; AC-74; Sigma; A5316), rabbit anti-PDGFRα (1:500; D1E1E; Cell Signaling; 3174), rabbit anti-FGFR1 (1:1,000; Abcam; EPR806Y), rabbit anti-IFT81 (1:500; Proteintech; 11744-1-AP), mouse anti-EB1 (1:500; BD Biosciences; 5/EB1), rabbit anti-IFT88 (1:500; Proteintech; 13967-1-AP), mouse anti-α-tubulin (1:1,000; 236-10501; A11126; Thermo Scientific), and mouse anti-vinculin (1:2,000; hVIN-1; Sigma; V9131). Secondary antibodies were horseradish peroxidase (HRP)-conjugated rabbit or mouse anti-IgG antibodies (1:2,000; Cell Signaling).

### Small Interfering RNA Gene Knockdown

Small interfering RNA (siRNA)-mediated IFT88 knockdown was carried out using two pooled sequences, 5′-CGACUAAGUGCCAGACUCAUU-3′ and 5′-CCGAAGCACUUAACACUUA-3′, previously described (Robert et al., 2007), when indicated, or a SMARTpool ON-TARGETplus siRNA (GE Dharmacon, Lafayette, CO). SCLT1 knockdown was achieved using a siGENOME Smartpool siRNA (GE Dharmacon). Kif7 was downregulated using a SMARTpool ON-TARGETplus siRNA (GE Dharmacon). Cells were transfected with Lullaby (Oz Biosciences, San Diego, CA) (three sequential transfections) or Lipofectamine RNAimax for the Smartpool (two sequential transfections). Non-targeting (control) siRNA was purchased from QIAGEN (#1027281).

### Plasmids and Transfections

The human full-length FLAG-Kif7 construct was a kind gift from Dr. Max Liebau (University of Cologne, Cologne, Germany). Cells were transfected with Lipofectamine 3000 (Thermo Scientific).

All lentiviruses were generated by transient co-transfection of 293T cells with packaging and envelope vectors using polyethylenimine (PEI) from Polysciences as a transfection reagent. The TRIPZ inducible human shIFT88 plasmid (GE Dharmacon) was used for stable IFT88 gene knockdown. H23 cells were selected for using 2 μg/mL puromycin.

### Cell Viability Assays

4,000 cells/well (2,000 cells/well for A204/DasR) were seeded into a 96-well plate (Greiner Bio-One, Kremsmunster, Austria) and incubated for 24 hr at 37°C, 5% CO_2_. After that, medium (5% FBS) containing drugs or vehicle controls was added to the cells and incubated for an additional 72 hr. Cell viability was measured using CellTiter-Glo (Promega), using a Victor X5 2030 Multilabel plate reader (Perkin Elmer). Cisplatin was obtained from Cayman Chemical Company, doxorubicin from LC Labs, and rapamycin from Calbiochem (San Diego, CA, USA). Additional growth assays were carried out using a ViCell Cell Viability Analyzer (Beckman Coulter). Briefly, 125,000 cells were seeded on 60-mm dishes in media containing 10% FCS. Cells were treated for 3–5 days with different drug concentrations in media containing no serum. Following treatment, viability was assessed using the trypan blue exclusion method. Each condition has been measured in triplicate.

### Caspase 3/7 Assay

4,000 cells/well were seeded into a 96-well plate (Greiner Bio-One, Kremsmunster, Austria) and incubated for 24 hr at 37°C, 5% CO_2_. After that, medium (5% FBS) containing drugs or vehicle controls was added to the cells and incubated for an additional 48 hr. Caspase 3/7 activity was measured using Caspase 3/7 Glo (Promega), with a Victor X5 2030 Multilabel plate reader (Perkin Elmer).

### Cell Cycle Analysis

To determine the cell cycle distribution, DNA content was assessed using propidium iodide (PI) staining. Cells were trypsinized and fixed in ice-cold 70% ethanol, and then stained with 20 μg/mL PI and 100 μg/mL RNAase A for 30 min. Samples were run using a BD LSR II flow cytometer (BD Biosciences) and FlowJo to analyze results.

### Hedgehog Pathway qRT-PCR

RNA was extracted using RNA mini kit (Thermo Scientific). Primers and TaqMan probes for detection of human Tata binding protein (TBP), GLI1, and PTCH1 were purchased as Assays-on-Demand from Applied Biosystems (TBP, Hs00427620_m1; GLI1, Hs01110766_m1; PTCH1, Hs00181117_m1). SuperScript III Platinum One-Step qRT-PCR System (Invitrogen) was used for the qPCR (PCR protocol: 15 min 50°C, 2 min 95°C, 30–50× 15 s 95°C and 1 min 60°C). The amount of amplicon generated during the PCR was measured using a QuantStudio 6 Flex Real-Time PCR System (Applied Biosystems). Each sample was run in triplicate; controls without reverse transcriptase gave no signal in all samples.

### Soft Agar Assay

Each well of a six-well dish was coated with 1 mL of base layer containing 0.6% agar (Sigma-Aldrich). Cells were dissociated and filtered through 30-μm filter and sub-cultured by layering 1 × 10^4^ viable cells in 1.5 mL of culture medium (5% FBS) containing 0.3% agar over replicate base layers. An upper layer of 2 mL of culture medium (5% FBS) was applied to each well and changed every 3 days. Colonies were counted using Gelcount (Oxford Optronix).

### Statistical Tests

Statistical analyses and samples sizes are specified in the figure legends. The error bars indicate either SD or SE (indicated).

## Supplemental Information

Document S1. Figures S1–S7 and Tables S1 and S2

Document S2. Article plus Supplemental Information

## References

[bib1] Awada G., Kourie H.R., Awada A.H. (2015). Novel mechanisms and approaches in the medical therapy of solid cancers. Discov. Med..

[bib2] Basten S.G., Giles R.H. (2013). Functional aspects of primary cilia in signaling, cell cycle and tumorigenesis. Cilia.

[bib3] Caspary T., Larkins C.E., Anderson K.V. (2007). The graded response to Sonic Hedgehog depends on cilia architecture. Dev. Cell.

[bib4] Cevik S., Hori Y., Kaplan O.I., Kida K., Toivenon T., Foley-Fisher C., Cottell D., Katada T., Kontani K., Blacque O.E. (2010). Joubert syndrome Arl13b functions at ciliary membranes and stabilizes protein transport in *Caenorhabditis elegans*. J. Cell Biol..

[bib5] Christensen S.T., Clement C.A., Satir P., Pedersen L.B. (2012). Primary cilia and coordination of receptor tyrosine kinase (RTK) signalling. J. Pathol..

[bib6] de Bruin E.C., Cowell C., Warne P.H., Jiang M., Saunders R.E., Melnick M.A., Gettinger S., Walther Z., Wurtz A., Heynen G.J. (2014). Reduced NF1 expression confers resistance to EGFR inhibition in lung cancer. Cancer Discov..

[bib7] Faião-Flores F., Alves-Fernandes D.K., Pennacchi P.C., Sandri S., Vicente A.L., Scapulatempo-Neto C., Vazquez V.L., Reis R.M., Chauhan J., Goding C.R. (2017). Targeting the hedgehog transcription factors GLI1 and GLI2 restores sensitivity to vemurafenib-resistant human melanoma cells. Oncogene.

[bib8] Garcia-Gonzalo F.R., Reiter J.F. (2012). Scoring a backstage pass: mechanisms of ciliogenesis and ciliary access. J. Cell Biol..

[bib9] Goetz S.C., Ocbina P.J., Anderson K.V. (2009). The primary cilium as a Hedgehog signal transduction machine. Methods Cell Biol..

[bib10] Han Y.G., Kim H.J., Dlugosz A.A., Ellison D.W., Gilbertson R.J., Alvarez-Buylla A. (2009). Dual and opposing roles of primary cilia in medulloblastoma development. Nat. Med..

[bib11] He M., Subramanian R., Bangs F., Omelchenko T., Liem K.F., Kapoor T.M., Anderson K.V. (2014). The kinesin-4 protein Kif7 regulates mammalian Hedgehog signalling by organizing the cilium tip compartment. Nat. Cell Biol..

[bib12] Huangfu D., Anderson K.V. (2005). Cilia and Hedgehog responsiveness in the mouse. Proc. Natl. Acad. Sci. USA.

[bib13] Ikegami K., Heier R.L., Taruishi M., Takagi H., Mukai M., Shimma S., Taira S., Hatanaka K., Morone N., Yao I. (2007). Loss of alpha-tubulin polyglutamylation in ROSA22 mice is associated with abnormal targeting of KIF1A and modulated synaptic function. Proc. Natl. Acad. Sci. USA.

[bib14] Kitai H., Ebi H., Tomida S., Floros K.V., Kotani H., Adachi Y., Oizumi S., Nishimura M., Faber A.C., Yano S. (2016). Epithelial-to-mesenchymal transition defines feedback activation of receptor tyrosine kinase signaling induced by MEK inhibition in KRAS-mutant lung cancer. Cancer Discov..

[bib15] Lauth M., Bergström A., Shimokawa T., Toftgård R. (2007). Inhibition of GLI-mediated transcription and tumor cell growth by small-molecule antagonists. Proc. Natl. Acad. Sci. USA.

[bib16] Lauth M., Bergström A., Shimokawa T., Tostar U., Jin Q., Fendrich V., Guerra C., Barbacid M., Toftgård R. (2010). DYRK1B-dependent autocrine-to-paracrine shift of Hedgehog signaling by mutant RAS. Nat. Struct. Mol. Biol..

[bib17] Li L., Grausam K.B., Wang J., Lun M.P., Ohli J., Lidov H.G., Calicchio M.L., Zeng E., Salisbury J.L., Wechsler-Reya R.J. (2016). Sonic Hedgehog promotes proliferation of Notch-dependent monociliated choroid plexus tumour cells. Nat. Cell Biol..

[bib18] Manchado E., Weissmueller S., Morris J.P., Chen C.C., Wullenkord R., Lujambio A., de Stanchina E., Poirier J.T., Gainor J.F., Corcoran R.B. (2016). A combinatorial strategy for treating KRAS-mutant lung cancer. Nature.

[bib19] Moser J.J., Fritzler M.J., Rattner J.B. (2009). Primary ciliogenesis defects are associated with human astrocytoma/glioblastoma cells. BMC Cancer.

[bib20] Moser J.J., Fritzler M.J., Rattner J.B. (2014). Ultrastructural characterization of primary cilia in pathologically characterized human glioblastoma multiforme (GBM) tumors. BMC Clin. Pathol..

[bib21] Neugebauer J.M., Amack J.D., Peterson A.G., Bisgrove B.W., Yost H.J. (2009). FGF signalling during embryo development regulates cilia length in diverse epithelia. Nature.

[bib22] O’Hagan R., Piasecki B.P., Silva M., Phirke P., Nguyen K.C., Hall D.H., Swoboda P., Barr M.M. (2011). The tubulin deglutamylase CCPP-1 regulates the function and stability of sensory cilia in *C. elegans*. Curr. Biol..

[bib23] Pazour G.J., Dickert B.L., Vucica Y., Seeley E.S., Rosenbaum J.L., Witman G.B., Cole D.G. (2000). *Chlamydomonas* IFT88 and its mouse homologue, polycystic kidney disease gene tg737, are required for assembly of cilia and flagella. J. Cell Biol..

[bib24] Pedersen L.B., Geimer S., Sloboda R.D., Rosenbaum J.L. (2003). The microtubule plus end-tracking protein EB1 is localized to the flagellar tip and basal bodies in *Chlamydomonas reinhardtii*. Curr. Biol..

[bib25] Plotnikova O.V., Pugacheva E.N., Golemis E.A. (2009). Primary cilia and the cell cycle. Methods Cell Biol..

[bib26] Robert A., Margall-Ducos G., Guidotti J.E., Bregerie O., Celati C., Brechot C., Desdouets C. (2007). The intraflagellar transport component IFT88/polaris is a centrosomal protein regulating G1-S transition in non-ciliated cells (vol 120, pg 628, 2006). J. Cell Sci..

[bib27] Saafan H., Foerster S., Parra-Guillen Z.P., Hammer E., Michaelis M., Cinatl J., Volker U., Frohlich H., Kloft C., Ritter C.A. (2016). Utilising the EGFR interactome to identify mechanisms of drug resistance in non-small cell lung cancer: proof of concept towards a systems pharmacology approach. Eur. J. Pharm. Sci..

[bib28] Sarkisian M.R., Siebzehnrubl D., Hoang-Minh L., Deleyrolle L., Silver D.J., Siebzehnrubl F.A., Guadiana S.M., Srivinasan G., Semple-Rowland S., Harrison J.K. (2014). Detection of primary cilia in human glioblastoma. J. Neurooncol..

[bib29] Schrøder J.M., Larsen J., Komarova Y., Akhmanova A., Thorsteinsson R.I., Grigoriev I., Manguso R., Christensen S.T., Pedersen S.F., Geimer S., Pedersen L.B. (2011). EB1 and EB3 promote cilia biogenesis by several centrosome-related mechanisms. J. Cell Sci..

[bib30] Sharma S.V., Lee D.Y., Li B., Quinlan M.P., Takahashi F., Maheswaran S., McDermott U., Azizian N., Zou L., Fischbach M.A. (2010). A chromatin-mediated reversible drug-tolerant state in cancer cell subpopulations. Cell.

[bib31] Tan D.S., Yom S.S., Tsao M.S., Pass H.I., Kelly K., Peled N., Yung R.C., Wistuba I.I., Yatabe Y., Unger M. (2016). The International Association for the Study of Lung Cancer Consensus Statement on Optimizing Management of EGFR Mutation-positive Non-small Cell Lung Cancer: status in 2016. J. Thorac. Oncol..

[bib32] Tanos B.E., Yang H.J., Soni R., Wang W.J., Macaluso F.P., Asara J.M., Tsou M.F. (2013). Centriole distal appendages promote membrane docking, leading to cilia initiation. Genes Dev..

[bib33] Vyse S., McCarthy F., Broncel M., Paul A., Wong J.P., Bhamra A., Huang P.H. (2018). Quantitative phosphoproteomic analysis of acquired cancer drug resistance to pazopanib and dasatinib. J. Proteomics.

[bib34] Wang J., Barr M.M. (2016). Ciliary extracellular vesicles: Txt Msg organelles. Cell. Mol. Neurobiol..

[bib35] Wong S.Y., Seol A.D., So P.L., Ermilov A.N., Bichakjian C.K., Epstein E.H., Dlugosz A.A., Reiter J.F. (2009). Primary cilia can both mediate and suppress Hedgehog pathway-dependent tumorigenesis. Nat. Med..

[bib36] Wong J.P., Todd J.R., Finetti M.A., McCarthy F., Broncel M., Vyse S., Luczynski M.T., Crosier S., Ryall K.A., Holmes K. (2016). Dual targeting of PDGFRα and FGFR1 displays synergistic efficacy in malignant rhabdoid tumors. Cell Rep..

[bib37] Wood C.R., Huang K., Diener D.R., Rosenbaum J.L. (2013). The cilium secretes bioactive ectosomes. Curr. Biol..

[bib38] Wu S.G., Liu Y.N., Tsai M.F., Chang Y.L., Yu C.J., Yang P.C., Yang J.C., Wen Y.F., Shih J.Y. (2016). The mechanism of acquired resistance to irreversible EGFR tyrosine kinase inhibitor-afatinib in lung adenocarcinoma patients. Oncotarget.

[bib39] Yeh A.C., Ramaswamy S. (2015). Mechanisms of cancer cell dormancy—another hallmark of cancer?. Cancer Res..

