# Supplementary material for: Primary Cilia Mediate Diverse Kinase Inhibitor Resistance Mechanisms in Cancer
Source: Cell Rep. 2018 Jun 8;23(10):3042–55. doi: 10.1016/j.celrep.2018.05.016 (PMC6016080; doi:10.1016/j.celrep.2018.05.016)
Supplement: Document S1. Figures S1–S7 and Tables S1 and S2 [file mmc1.pdf]

Cell Reports, Volume *23*

Supplemental Information

Primary Cilia Mediate Diverse Kinase Inhibitor

Resistance Mechanisms in Cancer

Andrew D. Jenks, Simon Vyse, Jocelyn P. Wong, Eleftherios Kostaras, Deborah Keller, Thomas Burgoyne, Amelia Shoemark, Athanasios Tsalikis, Maike de la Roche, Martin Michaelis, Jindrich Cinatl Jr., Paul H. Huang, and Barbara E. Tanos

Supplemental Information

Primary cilia mediate diverse kinase inhibitor resistance mechanisms in cancer

Andrew D. Jenks, Simon Vyse, Jocelyn P. Wong, Eleftherios Kostaras, Deborah Keller, Thomas Burgoyne, Amelia Shoemark, Athanasios Tsalikis, Maike de la Roche, Martin Michaelis, Jindrich Cinatl Jr, Paul H. Huang and Barbara E.Tanos

Supplementary Fig. 1

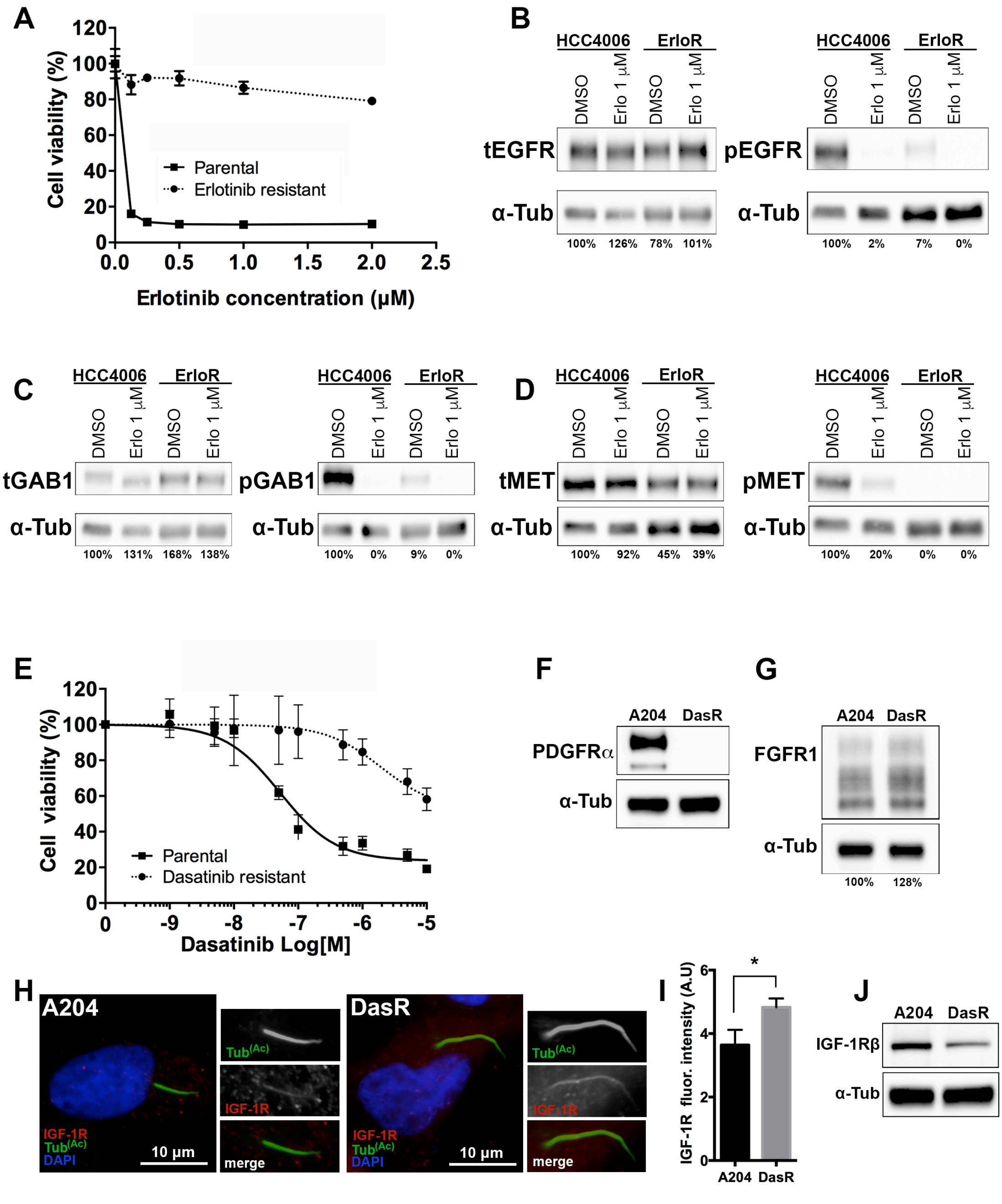

Supplementary figure 1. Molecular characterization of kinase-inhibitor-sensitive/kinase-inhibitor-resistant isogenic cell line pairs. ***Related to Figure 1.***
(**A**) Erlotinib dose response curve in parental and erlotinib-resistant HCC4006 cells. Cells were treated with a range of concentrations of erlotinib. Graph depicts cell viability normalized to DMSO control (n=3). (**B, C, D**) Western blots showing levels of total and phosphorylated EGFR (B), GAB1 (C) and MET (D) for HCC4006 parental and erlotinib resistant (ErloR) sublines. α-tubulin was used

as a loading control. Cells were treated with or without erlotinib (1 μM) for 6 hours. (**E**) Dasatinib dose response curve in parental and dasatinib-resistant A204 cells. Cells were treated with a range of dasatinib concentrations. Graph depicts cell viability normalized to DMSO control (n=3). (**F, G**) Western blots showing PDGFRα (**F**) and FGFR1 (**G**) of A204 and DasR cells (indicated). Note that DasR cells have no detectable PDGFRα and a slight increase in FGFR1 levels. (**H**) DasR cells show increased ciliary localization of IGF-1Rβ compared to control cells. A204 (left) or DasR cells (right) were serum starved for 48 hours to induce ciliogenesis. After fixation, cells were stained with acetylated tubulin (green), IGF-1Rβ (red) and DAPI (DNA). (**I**) Quantification of IGF-1Rβ cilia fluorescence intensities shown in **H**. Fluorescence intensities were normalized to background camera fluorescence intensity. n = 150 cilia, error bars represent s.d. $p<0.03$, unpaired T test. (**J**) Western blot showing total levels of IGF-1Rβ (upper panel, indicated) and loading controls (lower panel) in A204 and DasR cells.

Supplementary Fig. 2

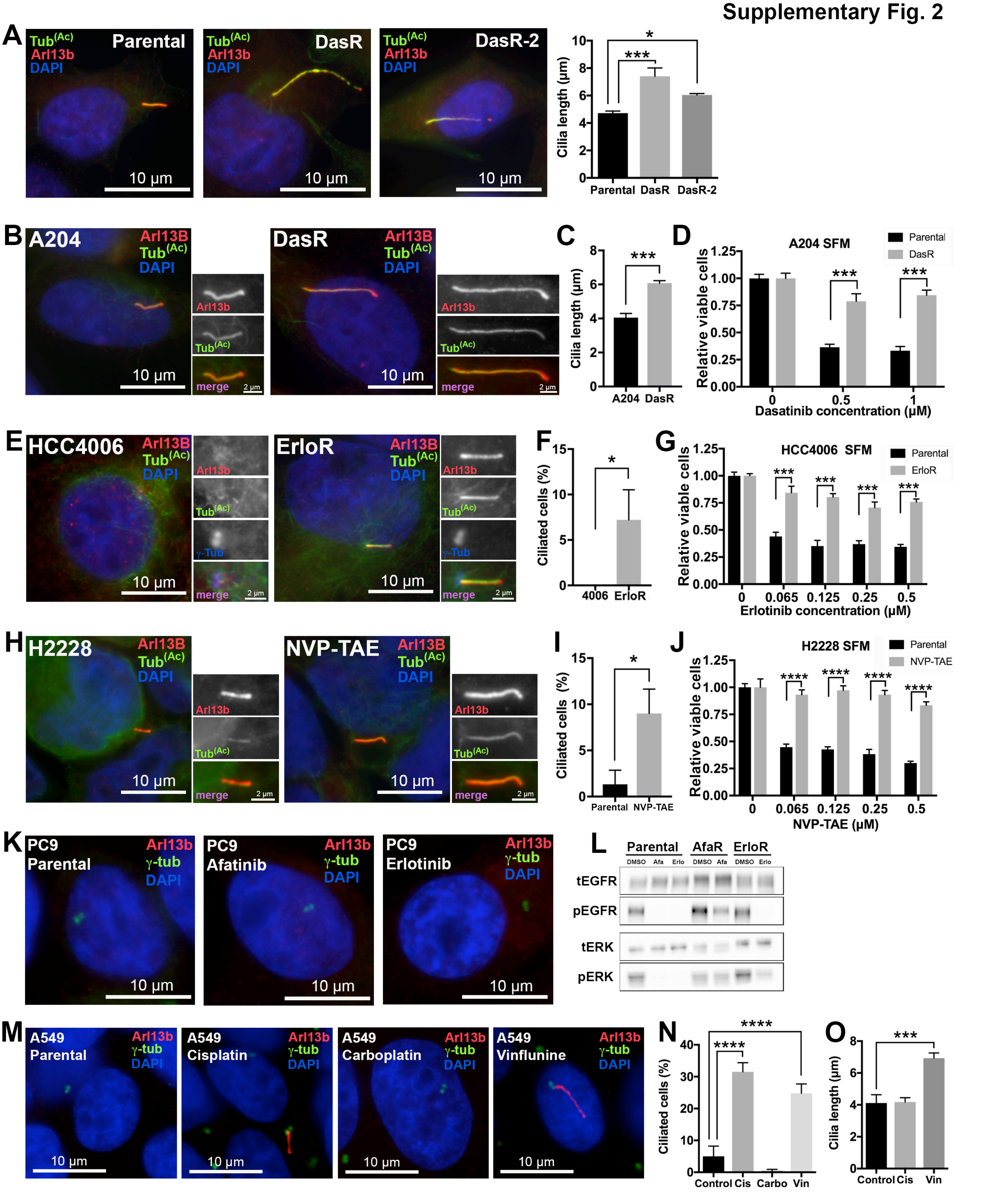

Supplementary figure 2. Ciliogenesis in additional models of drug resistance and growth conditions. *Related to Figure 1.* (**A**) Rhabdoid tumor A204 cells and two independently derived dasatinib resistant sublines (DasR and DasR-2) were stained with acetylated tubulin to mark cilia (green), Arl13b (red) and with DAPI (blue). Cilia length quantification is shown on the right. (n =

150), error bars represent the s.d. $p<0.0004$ (parental vs DasR) and $p<0.02$ (parental vs DasR-2) and, Tukey's multiple comparison test. (**B**) A204 cells (left panels), or a dasatinib resistant (DasR) subline (right panels) were grown in 5% FBS for 48 hours, then fixed and stained with antibodies for acetylated tubulin (green), Arl13B (red) to mark cilia and DAPI (blue). (**C**) Quantification of cilia length for cells shown in **(B)**. n = 150. Error bars represent s.d. $p<0.0003$, unpaired T test. (**D**) Analysis of cell viability in serum free conditions. A204 parental and DasR (indicated) cells after 72 hours of dasatinib treatment in serum free media (SFM). n = 3, p <0.009 for 0.5 μM, p<0.003 for 1 μM, unpaired T test. (**E**) Control (left panel) or erlotinib resistant (ErloR) (right panel) HCC4006 were grown in 5% FBS for 48hrs then stained with acetylated tubulin (green) and Arl13B (red) to mark cilia, γ-tubulin (blue/inset) and with DAPI (blue). Note that primary cilia were absent from HCC4006 cells but surprisingly are present in the erlotinib-resistant subline. (**F**) Quantification of ciliated cells from **(E)**. n = 300. Error bars represent s.d. $p<0.02$, unpaired T test. (**G**) Cell viability of HCC4006 parental and ErloR (indicated) cells after 72 hours of erlotinib treatment in serum free media (SFM). n = 3, p <0.002 (0.65 and 0.25 μM) 0.0009 (0.125 μM) 0.0002 (0.5 μM), unpaired T test. (**H**) NCI-H2228 parental (left panel) or NVP-TAE684 resistant subline (NVP-TAE684) (right panel) were grown in 5% FBS for 48hrs then stained with acetylated tubulin (green) and Arl13B (red) to mark cilia, and with DAPI (blue). (**I**) Quantification of ciliated cells from **(H)**. n = 300. Error bars represent s.d. $p<0.013$, unpaired T test. (**J**) Cell viability of H2228 parental and NVP-TAE684 resistant cells (indicated) after 72 hours of NVP-TAE treatment in serum free media (SFM). n = 3, p <0.0001 (0.065, 0.125, 0.25 and 0.5 μM), unpaired T test. (**K**) PC9 parental cells and sublines resistant to afatinib or erlotinib were stained with Arl13b to mark cilia (red), γ-tubulin (green) and with DAPI (blue). (**L**) Western blot showing phosphorylated EGFR, total ERFR, phosphorylated ERK and total ERK in PC9 parental cells, afatinib (AfaR) and erlotinib (ErloR) resistant sublines. Cells were treated with or without afatinib (2 μM) or erlotinib (1 μM) for 3 hours when indicated. (**M**) A549 parental cells and sublines resistant to cisplatin (cis), carboplatin (carbo) and vinflunine (vin) were stained with Arl13b to mark cilia (red), γ-tubulin (green) and with DAPI (blue). Note the increased ciliogenesis in sublines resistant to cisplatin and vinflunine. (**N, O**) Quantification of ciliated cells (**N**) and cilia length (**O**) shown in **M**. n = 300 (**N**), n = 150 (**O**). Error bars represent s.d. $p<0.0001$ (**N**), $p<0.004$ (**O**) Tukey's multiple comparison test.

Supplementary Fig. 3

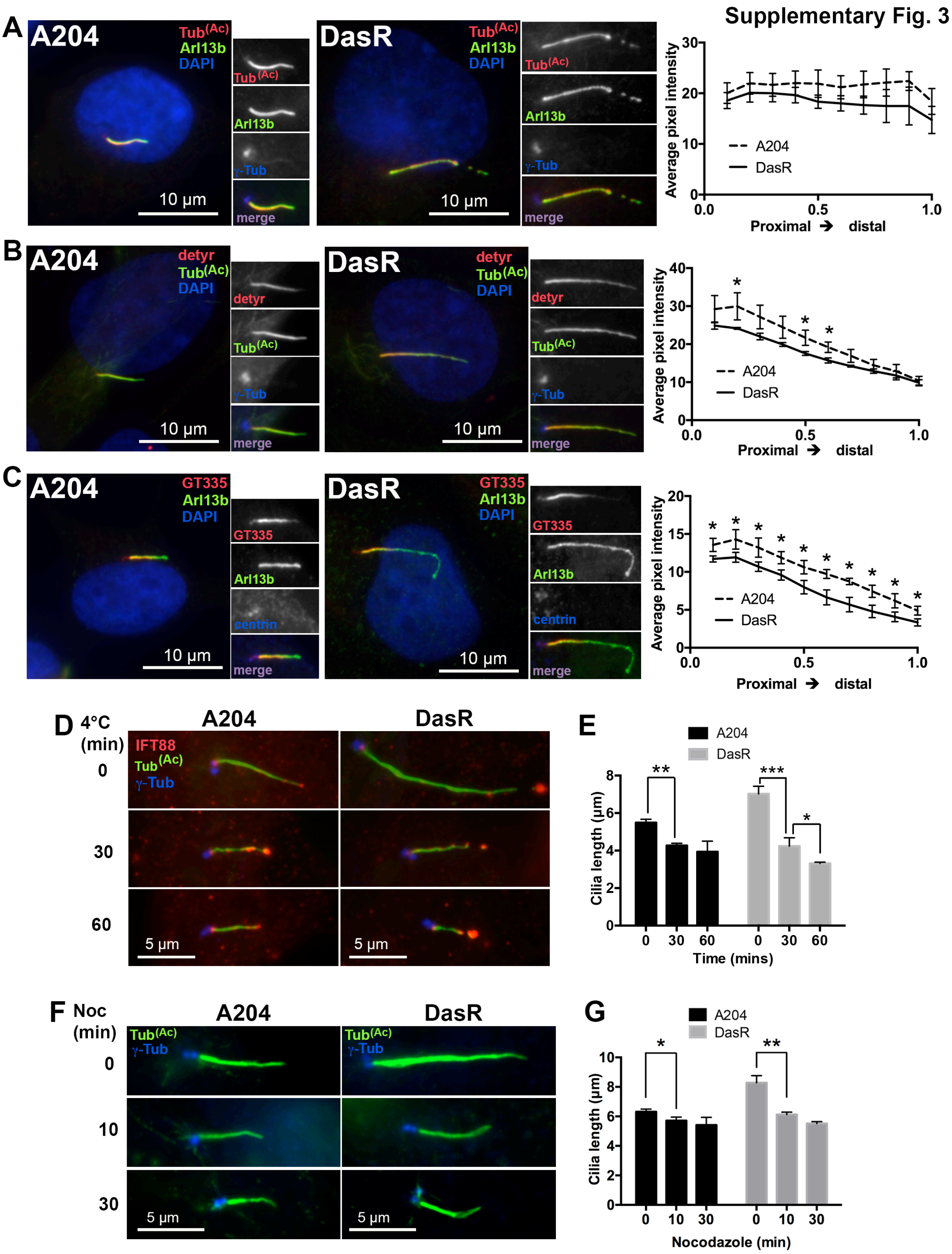

Supplementary figure 3. Cilia stability and tubulin posttranslational modifications in DasR cells. ***Related to Figure 2.*** (**A, B, C**) Control (left panels) or dasatinib resistant cells (right panels) were serum starved for 48 hours to induce cilia formation, then fixed and stained with antibodies to mark cilia (Arl13B or acetylated tubulin, indicated), together with antibodies for different post-translational modifications including acetylated tubulin (**A**) detyrosinated tubulin (**B**) and glutamylated tubulin (GT335) (**C**). Centrioles are marked in blue (insets) with γ-tubulin or centrin (indicated) and DAPI is shown in blue. Graphs on the right show a quantitative analysis of the results for **A**, **B** and **C**. n = 150, error bars represent s.d. Detyrosinated tubulin (**B**) P-values, proximal to distal: <0.05 <0,03, <0.02. Glutamylated tubulin (**C**) P-values, proximal to distal: <0.03, <0.04, <0.05, <0.03, <0.03, <0.01, <0.007, <0.02, <0.04, <0.03. Note that DasR cilia show less

glutamylated tubulin (GT335) along the axoneme compared to parental A204 cells. (**D**) Time course of cilia retraction in response to cold treatment (4°C) in A204 and DasR cells (indicated). Acetylated tubulin is shown in green, IFT88 in red and γ-tubulin in blue. (**E**) Quantification of cilia length in response to cold treatment for the experiment shown in **D**. Cilium length was measured using acetylated tubulin, n = 150. Error bars represent the s.d. $p<0.003$ between A204 at 0 and 30 mins, $p<0.0001$ between DasR at 0 and 30 mins and $p<0.02$ between DasR at 30 and 60 mins, Tukey's multiple comparison test. (**F**) Time course of cilia retraction in response to nocodazole (10 μM) in A204 (left) and DasR cells (right). Acetylated tubulin staining marks primary cilia (green) and γ-tubulin marks centrioles (blue). (**G**) Cilium length was measured using acetylated tubulin staining from the experiment shown in **F**. n = 150 cilia, error bars represent the s.d. $p<0.02$ for A204 0 and 10 mins and $p<0.0001$ DasR 0 and 10 mins, Tukey's multiple comparison test.

Supplementary Fig. 4

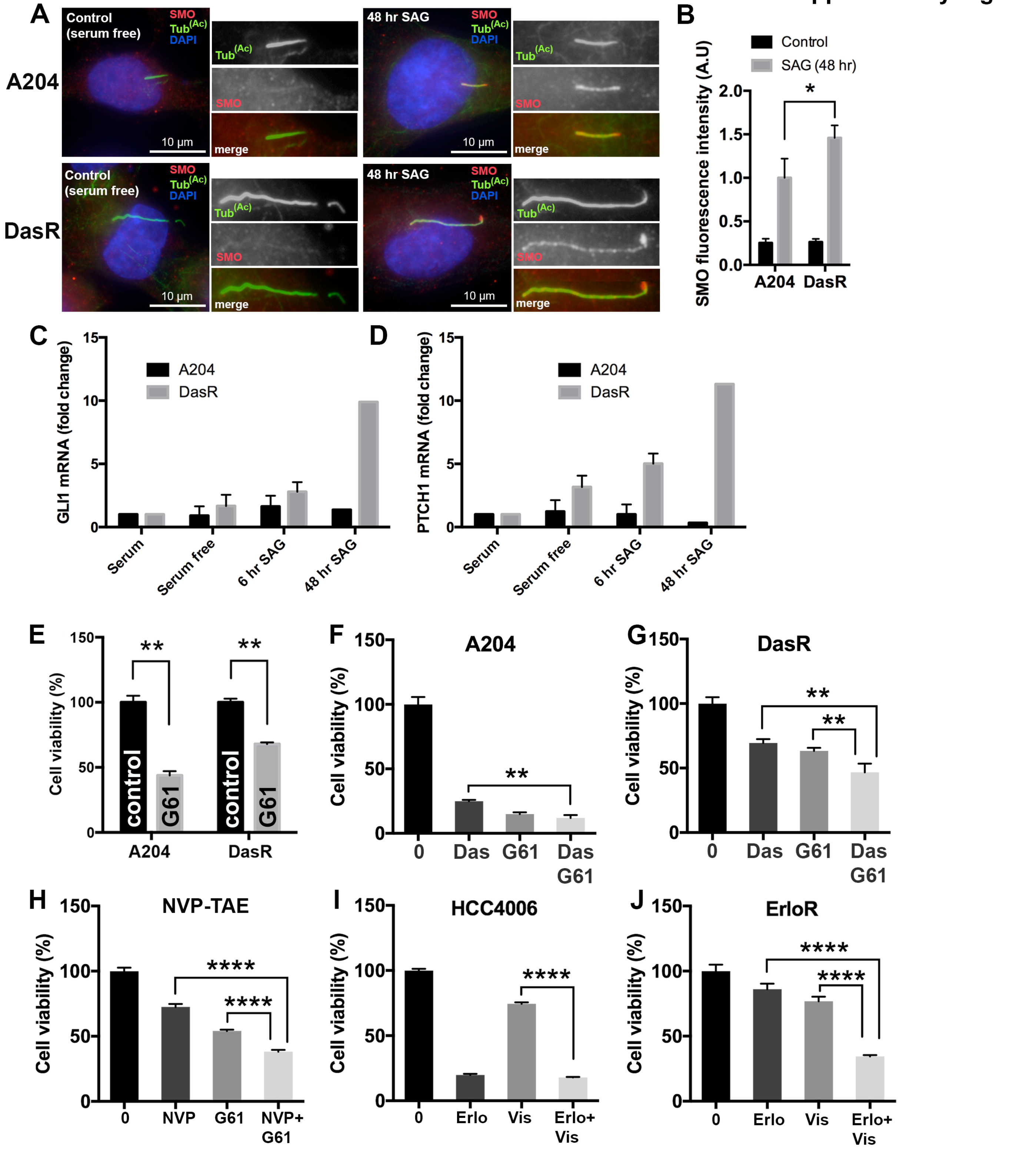

Supplementary figure 4. Kinase inhibitor resistant cells show increased Hh pathway activation and are sensitive to Hh pathway inhibition. *Related to Figure 3.* (**A**) A204 cells (top panel), or a dasatinib resistant (DasR) subline (lower panel) were serum starved for 24 hours and either left untreated for an additional 48 hours (left) or treated with SAG (100 nM) for the same amount of time (right). Cells were then fixed and stained with antibodies for acetylated tubulin to mark cilia (green), SMO (red) and with DAPI (blue) to mark DNA. (**B**) Quantification of SMO cilia fluorescence intensities for the experiment shown in **A**. Note the increased SMO fluorescence intensity in DasR compared to A204. Fluorescence intensity was normalized to surrounding fluorescence, n = 150, error bars represent s.d. p<0.04, unpaired T test. (**C, D**) Quantitative polymerase chain reaction (qPCR) showing fold change (relative to no serum starvation) mRNA levels of GLI1 (**C**) and PTCH1 (**D**) in A204 and DasR cells. Note the fold change of both *GLI1* (**C**)

and *PTCH1* (**D**) is increased in DasR cells compared to A204 at all time points. GLI1 and PTCH1 mRNA values are normalized to TATA box–binding protein (*TBP*) mRNA values, fold change calculated by comparing to mRNA levels prior to serum starvation; n = 3 (0-6h). (**E**) Cell viability of A204 and DasR cells (indicated), in normal media (black columns) or with the addition Hh pathway inhibitor GANT61 (G61) (2.5 μM) (grey columns). Cell viability was normalized to DMSO control treated cells. p<0.01 unpaired T test. (**F**) Cell viability of A204 with the addition of GANT61 (G61) (10 μM), dasatinib (5 μM) or a combination of both. Cell viability was normalized to DMSO control treated cells. p<0.005 Tukey's multiple comparison test. (**G**) Cell viability of DasR with the addition of GANT61 (G61) (10 μM), dasatinib (5 μM) or a combination of both. Cell viability was normalized to DMSO control treated cells. p<0.002 (Das vs Das+G61), p<0.001 (G61 vs Das+G61) Tukey's multiple comparison test. (**H**) Cell viability (Cell titer Glo) of NCI-H2228 NVP-TAE684 resistant subclone treated with GANT61 (G61) (20 μM), NVP-TAE684 (0.5 μM) (NVP), or a combination of both. n = 3, cell viability is normalized to the DMSO control. Error bars represent s.d. p<0.0001 (NVP-TAE684 vs NVP-TAE684 +GANT61 and GANT61 vs NVP-TAE684 +GANT61), Tukey's multiple comparison test. (**I**) Cell viability (Cell titer Glo) of HCC4006 treated with vismodegib (vis) (40 μM), erlotinib (0.5 μM), or a combination of both. n = 3, cell viability is normalized to the DMSO control. Error bars represent s.d. p<0.0001, Tukey's multiple comparison test. (**J**) Cell viability (Cell titer Glo) of ErloR cells treated with vismodegib (vis) (40 μM), erlotinib (0.5 μM), or a combination of both. n = 3, cell viability is normalized to the DMSO control. Error bars represent s.d. p<0.0001 (Erlo vs Erlo+vis and vis vs Erlo+vis), Tukey's multiple comparison test. Note that the double treatment significantly sensitizes ErloR cells to erlotinib.

Supplementary Fig. 5

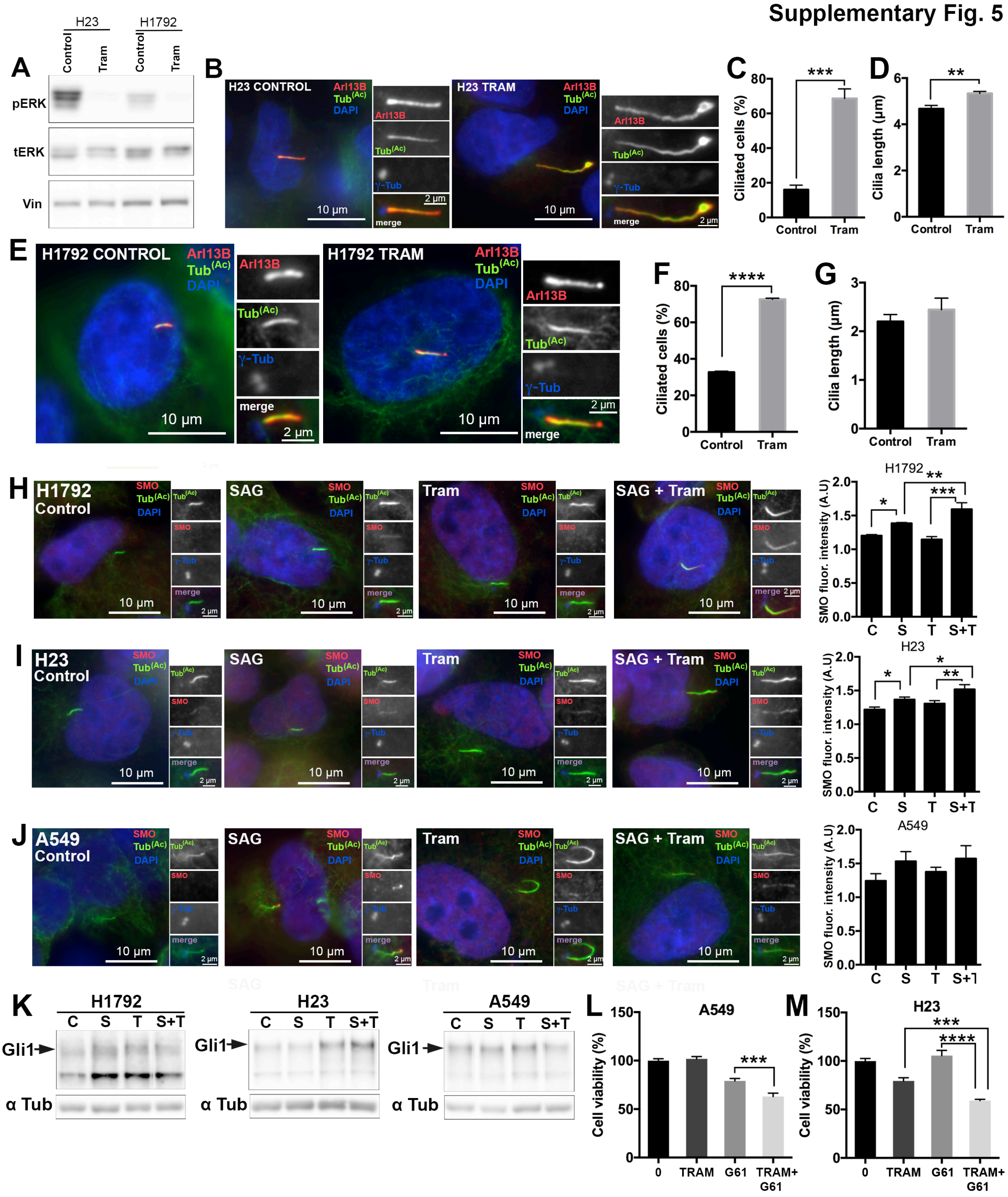

Supplementary figure 5. Mutant-KRAS NSCLC cells show increased cilia frequency and hedgehog pathway activation following MEK inhibitor treatment. ***Related to Figure 5.*** (**A**) Western blots showing NCI-H23 and NCI-H1792 pERK levels in the absence or presence of 50 nM trametinib (48 hrs). (**B**) NCI-H23 cells treated with 50 nM Trametinib or DMSO (control) for 48hrs then fixed and stained with antibodies for acetylated tubulin (green), Arl13B (red), γ-tubulin (blue/inset) and DAPI (blue). Note that exposure to trametinib promoted an increase in cilia length. (**C, D**) Quantification of ciliated cells (**C**) and cilia length (**D**) in **B**. n = 300 cells (**C**), n = 150 cilia (**D**), error bars represent the s.d. $p<0.0001$ (**C**) and $p<0.003$ (**D**), for an unpaired T test. (**E**) NCI-H1792 cells treated with 50 nM Trametinib or DMSO (control) for 48hrs then fixed and stained with antibodies for acetylated tubulin (green), Arl13B (red), γ-tubulin (blue/inset) and DAPI (blue). (**F, G**) Quantification of ciliated cells (**F**) and cilia length (**G**) shown in **E**. n = 300 cells (**F**), n = 150 cilia (**G**), error bars represent the s.d. $p<0.0001$ (**F**), for an unpaired T test. (**H**) NCI-H1792 cells were treated either with DMSO control (C), 100 nM SAG (S), 50 nM trametinib (T) or a combination of SAG and trametinib (S+T) for 48 hours. Cells were then fixed and stained with antibodies for

acetylated tubulin to mark cilia (green), SMO (red) and with DAPI (blue) to mark DNA. Quantification of SMO cilia fluorescence intensities is shown on the right. Note the combination of trametinib and SAG increases SMO cilia fluorescence compared to SAG alone. Fluorescence intensity was normalized to surrounding fluorescence, n = 150, error bars represent s.d. $p<0.02$ (C vs S), $p<0.0001$ (T vs S+T), $p<0.007$ (S vs S+T), Tukey's multiple comparison test. (**I**) NCI-H23 cells were treated either with DMSO control (C), 100 nM SAG (S), 50 nM trametinib (T) or a combination of SAG and trametinib (S+T) for 48 hours. Cells were then fixed and stained with antibodies for acetylated tubulin to mark cilia (green), SMO (red) and with DAPI (blue) to mark DNA. Quantification of SMO cilia fluorescence intensities is shown on the right. Fluorescence intensity was normalized to surrounding fluorescence, n = 150, error bars represent s.d. $p<0.03$ (C vs S), $p<0.004$ (T vs S+T), $p<0.03$ (S vs S+T), Tukey's multiple comparison test. (**J**) A549 cells were treated either with DMSO control (C), 100 nM SAG (S), 50 nM trametinib (T) or a combination of SAG and trametinib (S+T) for 48 hours. Cells were then fixed and stained with antibodies for acetylated tubulin to mark cilia (green), SMO (red) and with DAPI (blue) to mark DNA. Quantification of SMO cilia fluorescence intensities is shown on the right. Fluorescence intensity was normalized to surrounding fluorescence, n = 150, error bars represent s.d. (**K**) Western blots showing GLI1 levels for H1792, H23 and A549 cells after 48hrs of DMSO (control) (C), SAG (S), Trametinib (T) or SAG and Trametinib (S+T) exposure. (**L**) Cell viability (Cell titer Glo) of A549 treated with GANT61 (G61) (5 μM), trametinib (1.56 nM), or a combination of both. n = 3, cell viability is normalized to the DMSO control. Error bars represent s.d. $p<0.0005$, Tukey's multiple comparison test. (**M**) Cell viability (Cell titer Glo) of H23 treated with GANT61 (G61) (5 μM), trametinib (1.56 nM), or a combination of both. n = 3, cell viability is normalized to the DMSO control. Error bars represent s.d. $p<0.0005$ (Tram vs Tram+G61), $p<0.0001$ (G61 vs Tram+G61), Tukey's multiple comparison test.

Supplementary Fig. 6

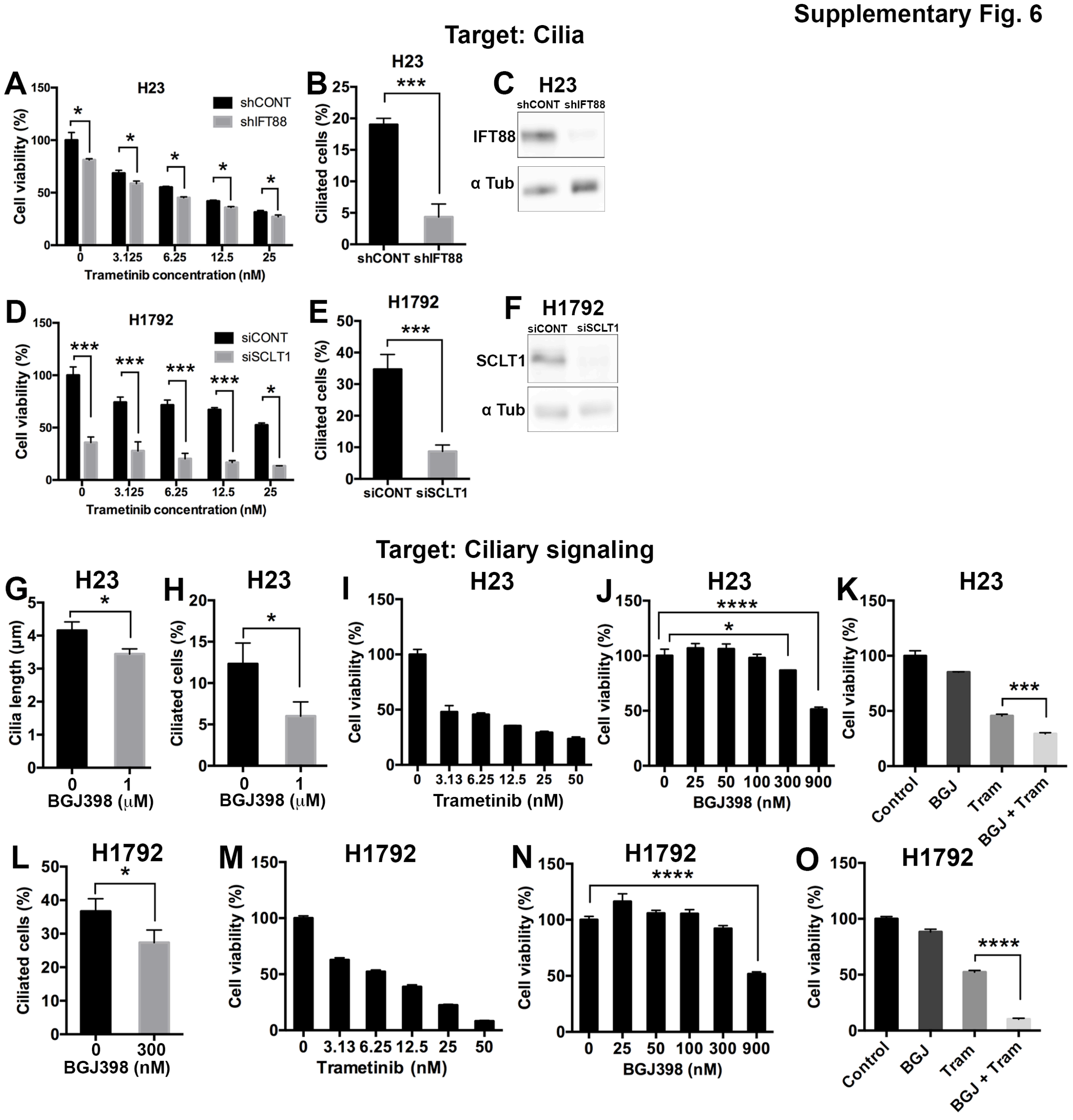

Supplementary figure 6. Cilia and ciliary pathways can mediate *de novo* drug resistance. *Related to Figures 4 and 5.* (**A**) Cell viability in H23 cells after treatment with the MEK inhibitor trametinib in control cells (shCONT) or upon downregulation of IFT88 with an inducible IFT88 shRNA (shIFT88). Cell viability was normalized to shCONT (DMSO). n = 4, p<0.003 for 0 nM, p<0.002 for 3.125 nM, p<0.0001 for 6,25 nM, p<0.0001 for 12.5 nM and p<0.001 for 25 nM, unpaired T test. (**B**) Quantification of percent ciliated cells (n=300) for the experiment shown in **A**. Error bars represent s.d. p<0.0005, unpaired T test. (**C**) Western blot showing IFT88 levels in H23 cells virally transduced with control shRNA (shCONT) or IFT88 shRNA (shIFT88) for the experiments shown in **A, B**. **(D**) Cell viability in H1792 cells after treatment with the MEK inhibitor trametinib in control cells (siCONT) or upon down-regulation of SCLT1 (siSCLT1). Cell viability was normalized to siCONT (DMSO), p<0.0004 for 0 nM, p<0.002 for 3.125 nM, p<0.0003 for 6,25 nM, p<0.0001 for 12.5 nM and p<0.0001 for 25 nM, unpaired T test. (**E**) Quantification of percent ciliated cells (n=300) for the experiment shown in **D**. Error bars represent s.d. p<0.002, unpaired T test. (**F**) Western blot showing SCLT1 levels in H1792 cells transfected with siCONT or siSCLT1 (indicated) for the experiments shown in **D, E**. (**G**) Cilia length quantification of H23 cells treated with or without the FGFR inhibitor BGJ398 for 48 hours. Note that after treatment with BGJ398 cilia length was reduced. n = 150, error bars represent s.d. p<0.02, unpaired T test. (**H**) Cilia percentage quantification of H23 cells when treated with or without the FGFR inhibitor BGJ398 for

48 hours. Note that after treatment with BGJ398 cilia percentage was reduced. n = 150, error bars represent s.d. $p<0.03$, unpaired T test. (**I**) Cell viability (Cell titer Glo) of H23 grown in a range of trametinib concentrations. Cell viability was normalized to DMSO control (n=3). Error bars represent s.d. (**J**) Cell viability (Cell titer Glo) of H23 grown in a range of concentrations of the FGFR inhibitor BGJ398. Cell viability was normalized to DMSO control (n=3). Error bars represent s.d. $p<0.02$ (0 nM vs 300 nM), $p<0.0001$ (0 nM vs 900 nM), Tukey's multiple comparison test. (**K**) Cell viability (Cell titer Glo) of H23 cells treated with trametinib (6.25 nM), BGJ398 (300 nM), or both. n = 3, cell viability is normalized to the DMSO control. Error bars represent s.d. $p<0.0003$, for an unpaired T test. (**L**) Cilia percentage quantification of H1792 cells treated with or without BGJ398 for 48 hours. Note that after treatment with BGJ398 cilia percentage was reduced. n = 150, error bars represent s.d. $p<0.04$, unpaired T test. (**M**) Cell viability (Cell titer Glo) of H1792 grown in a range of trametinib concentrations. Cell viability was normalized to DMSO control (n=3). Error bars represent s.d. (**N**) Cell viability (Cell titer Glo) of H1792 grown in a range of concentrations of BGJ398. Cell viability was normalized to DMSO control (n=3). Error bars represent s.d. $p<0.0001$, Tukey's multiple comparison test. (**O**) Cell viability (Cell titer Glo) of H1792 cells treated with trametinib (6.25 nM), BGJ398 (300 nM), or both. n = 3, cell viability is normalized to the DMSO control. Error bars represent s.d. $p<0.0001$, for an unpaired T test.

Supplementary Fig. 7

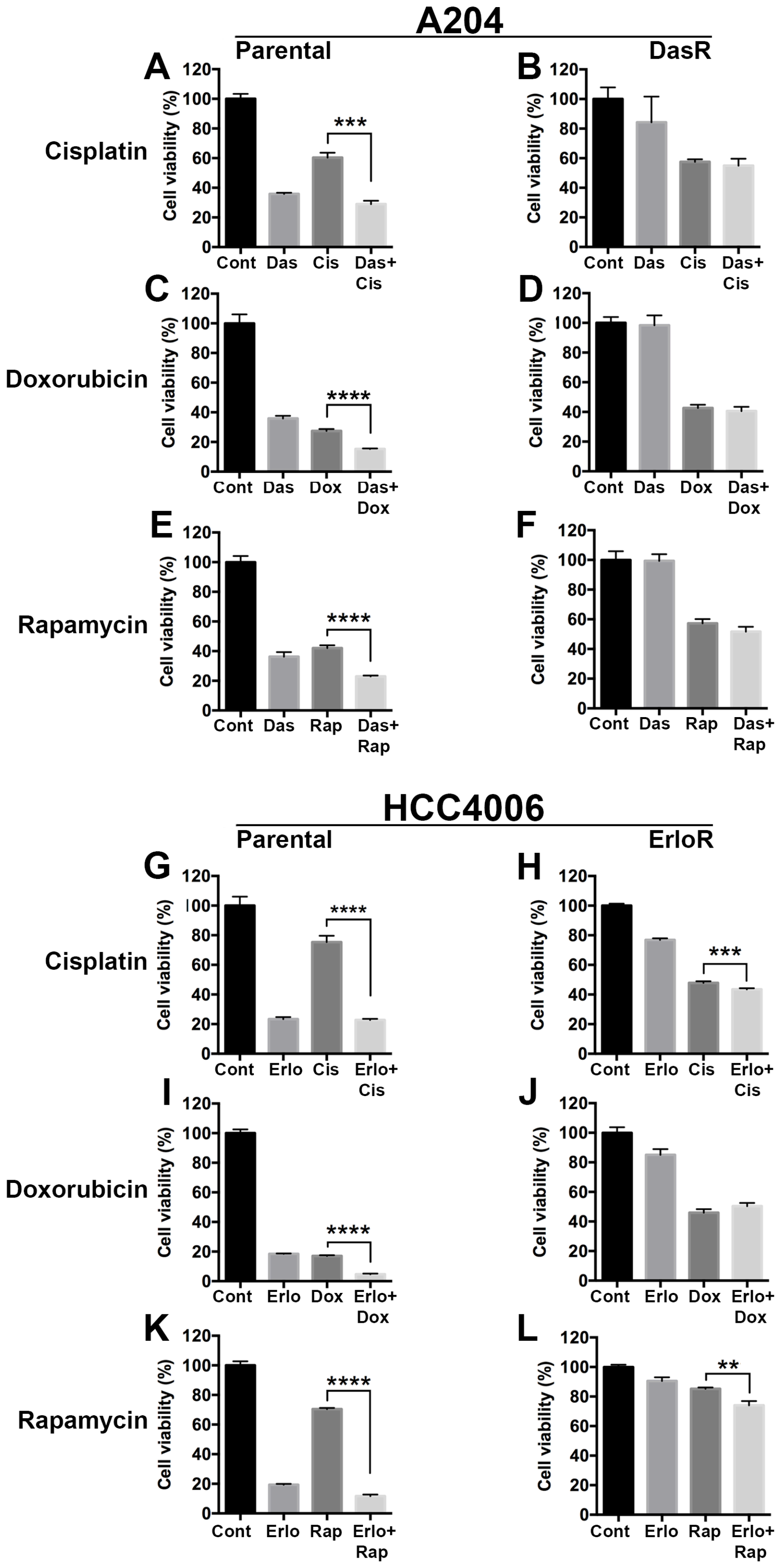

Supplementary figure 7. Cell cycle arrest does not sensitize kinase inhibitor resistant cells to kinase inhibitors. ***Related to Figures 1 and 4.*** (**A, B**) Cell viability (Cell titer Glo) of A204 (**A**) and DasR (**B**) cells treated with the S phase cell cycle inhibitor cisplatin (1 μM), dasatinib (0.5 μM), or both. n = 3, cell viability is normalized to the DMSO control. Error bars represent s.d. $p<0.0002$ (**A**), unpaired T test. (**C, D**) Cell viability (Cell titer Glo) of A204 (**C**) and DasR (**D**) cells treated with the G2/M phase cell cycle inhibitor doxorubicin (0.1 μM), dasatinib (0.5 μM), or both. n = 3, cell viability is normalized to the DMSO control. Error bars represent s.d. $p<0.0001$ (**C**), unpaired T test. (**E, F**) Cell viability (Cell titer Glo) of A204 (**E**) and DasR (**F**) cells treated with the G1 phase

cell cycle inhibitor rapamycin (0.5 μM), dasatinib (0.5 μM), or both. n = 3, cell viability is normalized to the DMSO control. Error bars represent s.d. $p<0.0001$ (**E**), unpaired T test. (**G, H**) Cell viability (Cell titer Glo) of HCC40006 parental (**G**) and ErloR (**H**) cells treated with the S phase cell cycle inhibitor cisplatin (15 μM), erlotinib (0.5 μM), or both. n = 4, cell viability is normalized to the DMSO control. Error bars represent s.d. $p<0.0001$ (**G**), $p<0.0006$ (**H**), unpaired T test. (**I, J**) Cell viability (Cell titer Glo) of HCC40006 parental (**I**) and ErloR (**J**) cells treated with the G2/M phase cell cycle inhibitor doxorubicin (1 μM), erlotinib (0.5 μM), or both. n = 3, cell viability is normalized to the DMSO control. Error bars represent s.d. $p<0.0001$ (**I**), unpaired T test. (**K, L**) Cell viability (Cell titer Glo) of HCC40006 parental (**K**) and ErloR (**L**) cells treated with the G1 phase cell cycle inhibitor rapamycin (1 μM), erlotinib (0.5 μM), or both. n = 3, cell viability is normalized to the DMSO control. Error bars represent s.d. $p<0.0001$ (**K**), $p<0.004$ (**L**) unpaired T test.

Supplementary Table 1

Asynchronously cycling cells treated with control siRNA (siCONT) or siRNA for protein knockdown (siTARGET)

Cell line (siControl vs siTarget)	G0/G1 (%)		G2/M (%)		S (%)	
	siCont	siTarget	siCont	siTarget	siCont	siTarget
A204 (siCONT/siIFT88)	64.9	71.0	23.3	20.2	5.50	4.79
A204 DasR (siCONT/siIFT88)	81.3	86.4	13.0	9.95	3.86	2.42
A549 (siCONT/siIFT88)	67.7	72.1	15.1	15.2	15.4	11.4
A549 (siCONT/siSCLT1)	67.7	75.5	15.1	9.85	15.4	11.5
H23 (shSCLT1/shIFT88)	62.2	62.6	24.4	24.9	11.0	8.98
HCC4006 parental (siCONT/siSCLT1)	87.5	85.2	3.12	2.40	8.29	10.7
HCC4006 ErloR (siCONT/siSCLT1)	73.0	70.5	15.1	15.6	11.0	13.0
A204 parental (siCONT/siKif7)	68.2	69.2	11.3	15.2	19.4	14.9
A204 DasR (siCONT/siKif7)	83.1	84.3	9.52	9.97	5.55	4.13

Asynchronously cycling cells grown with either DMSO (control) or a kinase inhibitor (14hr)

Cell line (DMSO control vs drug)	G0/G1 (%)		G2/M (%)		S (%)	
	DMSO	Drug	DMSO	Drug	DMSO	Drug
A204 parental (DMSO/dasatinib 5 μM)	70.3	81.9	20.1	14.1	5.57	1.95
A204 DasR (DMSO/dasatinib 5 μM)	80.0	77.3	14.2	14.9	3.90	5.37
A204 parental (DMSO/BGJ398 100 nM)	78.3	86.1	11.2	10.5	9.64	2.92
A204 DasR (DMSO/BGJ398 100 nM)	86.2	94.5	7.18	3.01	6.18	2.23
HCC4006 parental (DMSO/erlotinib 1 μM)	81.6	87.8	10.3	8.54	6.04	2.44
HCC4006 ErloR (DMSO/erlotinib 1 μM)	71.2	70.5	18.3	19.2	8.41	7.81
H2228 parental (DMSO/NVP-TAE684 0.5 μM)	72.4	81.3	12.8	11.5	13.6	6.14
NVP TAE resistant (DMSO/NVP-TAE684 0.5 μM)	79.7	83.3	10.3	7.32	9.44	8.87

Serum starved cells treated with either DMSO (control) or a kinase inhibitor (14hr)

Cell line (DMSO control vs drug)	G0/G1 (%)		G2/M (%)		S (%)	
	DMSO	Drug	DMSO	Drug	DMSO	Drug
H2228 parental (DMSO/NVP-TAE684 0.5 µM)	62.8	67.8	13.4	10.3	15.7	15.8
NVP-TAE resistant (DMSO/NVP-TAE684 0.5 µM)	75.4	76.5	14.9	14.0	6.50	6.27
A204 parental (DMSO/dasatinib 5 µM)	89.7	89.9	6.46	6.95	3.23	2.64
A204 DasR (DMSO/dasatinib 5 µM)	90.9	88.5	3.88	3.83	4.43	6.85
HCC4006 ErloR (DMSO/erlotinib 1 µM)	84.9	85.4	7.39	8.60	6.73	5.18
HCC4006 ErloR (DMSO/BGJ398 300 nM)	84.9	88.2	7.39	6.87	6.73	4.14

Serum starved (48 hr) parental and acquired kinase inhibitor resistant sublines

Cell line (parental vs resistant)	G0/G1 (%)		G2/M (%)		S (%)	
	Parental	Resistant	Parental	Resistant	Parental	Resistant
A204 serum starved (A204/DasR)	76.2	84.5	17.7	10.6	3.59	3.79
HCC4006 serum starved (HCC4006/ErloR)	85.2	85.1	8.57	10.1	5.03	3.32
H2228 serum starved (H2228/NVP-TAE684)	78.1	84.5	9.40	10.3	11.9	3.69

Supplementary Table 1. Cell cycle distributions for experiments shown*. Related to Figures 1, 2 and 4.* Cell cycle profiles of serum starved or asynchronously growing cells with the addition of kinase inhibitors or knockdown of SCLT1, IFT88 or Kif7. Note the minimal increase in cells in G0/G1 in A204 DasR, H23 and HCC4006 ErloR in response to siIFT88 compared siCONT. In addition siSCLT1 (A549) and siKif7 (A204) had minimal impact upon the cell cycle. Kinase inhibitors were tested in isogenic pairs, note the resistant sublines (HCC4006 ErloR, A204 DasR and H2228 NVP-TAE684 resistant) had minimal changes to their cell cycle in response to their corresponding drugs (compared to DMSO controls).

Supplementary Table 2.

A204 parental vs Dasatinib resistant (DasR)

**Cell line (time after serum re-challenge)**	**G0/G1 (%)**		**G2/M (%)**		**S (%)**	
	**Parental**	**DasR**	**Parental**	**DasR**	**Parental**	**DasR**
A204 (0hr- serum starved 48hr)	73.9	82.1	17.6	13.7	7.39	3.98
A204 (12hr)	77.8	84.8	11.3	8.21	8.82	6.60
A204 (24hr)	63.7	69.5	19.5	19.4	14.4	10.8
A204 (36hr)	62.8	78.0	16.8	9.40	17.0	11.8
A204 (48hr)	75.5	82.6	13.9	6.71	8.46	10.2

HCC4006 parental vs Erlotinib resistant (ErloR)

**Cell line (time after serum re-challenge)**	**G0/G1 (%)**		**G2/M(%)**		**S (%)**	
	**Parental**	**ErloR**	**Parental**	**ErloR**	**Parental**	**ErloR**
HCC4006 (0hr- serum starved 48hr)	83.4	82.2	7.16	11.2	7.70	5.90
HCC4006 (12hr)	88.4	83.1	7.77	12.2	2.63	3.83
HCC4006 (24hr)	64.6	41.2	12.0	31.1	20.3	24.2
HCC4006 (36hr)	74.7	80.6	14.4	13.7	9.03	4.66
HCC4006 (48hr)	87.7	87.4	6.29	8.24	3.78	3.52

H2228 parental vs NVP-TAE684 resistant (NVP-TAE)

**Cell line (time after serum re-challenge)**	**G0/G1 (%)**		**G2/M (%)**		**S (%)**	
	**Parental**	**NVP-TAE**	**Parental**	**NVP-TAE**	**Parental**	**NVP-TAE**
H2228 (0 hr- serum starved 48hr)	86.2	89.7	7.14	7.28	5.65	2.41
H2228 (12hr)	85.3	92.0	6.50	5.62	7.07	1.85
H2228 (24hr)	70.1	82.8	13.8	3.90	14.3	9.32
H2228 (36hr)	77.4	38.4	9.70	34.6	11.7	23.4
H2228 (48hr)	82.3	80.9	7.79	10.9	8.73	6.70

Supplementary Table 2. Kinetic analysis of cell cycle changes following serum starvation and re-challenge in isogenic parental and drug-resistant cell line pairs. ***Related to Figure 1.*** Cell lines were serum starved for 48 hours, and then grown in 10 % FBS for 48 hours. Cell cycle distributions were assessed every 12 hours.
